# Learning speed is affected by personality and reproductive investment in a songbird

**DOI:** 10.1371/journal.pone.0185410

**Published:** 2017-10-11

**Authors:** Hector Fabio Rivera-Gutierrez, Tine Martens, Rianne Pinxten, Marcel Eens

**Affiliations:** 1 Grupo Ecología y Evolución de Vertebrados, Instituto de Biología, Facultad de Ciencias Exactas y Naturales, Universidad de Antioquia UdeA, Medellin, Colombia; 2 University of Antwerp, Behavioural Ecology and Ecophysiology Group, Campus Drie Eiken, Universiteitsplein 1, Wilrijk, Belgium; 3 University of Antwerp, Faculty of Social Sciences, Research unit Didactica, Venusstraat 35, Antwerp; Liverpool John Moores University, UNITED KINGDOM

## Abstract

Individuals from different taxa, including songbirds, differ consistently in behaviour and personality when facing different situations. Although our understanding of animal behaviour has increased, knowledge about between-individual differences in cognitive abilities is still limited. By using an experimental approach and a free-living songbird (*Parus major*) as a model, we attempted to understand between-individual differences in habituation to playbacks (as a proxy of learning speed), by investigating the role of personality, age and reproductive investment (clutch size). Pre-breeding males were tested for exploration (a proxy of personality) in standardized conditions. In addition, the same individuals were exposed to three playbacks in the field during incubation. Birds significantly moved less, stayed further away and overlapped less the playback with successive playback stimulation. While a decrease in the locomotor behaviour can be explained by personality, differences in habituation of overlapping were predicted by both reproductive investment and personality. Fast explorers habituated less. Moreover, males paired to females with larger clutches did not vary the intensity of overlapping. Since habituation requires information for recognition of non-threatening signals, personality may bias information gathering. While fast explorers may collect less information from the environment, slow explorers (reactive birds) seem to pay attention to environmental clues and collect detailed information. We provided evidence that the rate of habituation of behavioural responses, a proxy of cognitive abilities, may be affected by different factors and in a complex way.

## Introduction

Consistent between-individual differences in behaviour throughout time and across different contexts have been reported for several taxa including songbirds, reptiles, fishes, insects and mammals [1–8], and have been mainly studied in the framework of personality, behavioural syndromes or coping styles [1, 9–11]. Personality in the framework of animal behaviour is usually defined as a suite of correlated behaviours that are expressed consistently across different situations [11, 12]. Stating that correlated behaviours are consistent, implies that rank-order differences between individuals through time and/or across contexts must remain the same [11]. Correlations between behaviours have important ecological and evolutionary implications, because they are linked to different trade-offs [11]. For example, a suite of correlated behaviours may favour reproductive success under particular circumstances, but fluctuating environments may change selection pressures, thus helping to maintain individual variation in behaviour, which may in turn have fitness costs or benefits [11, 13].

Personality or behavioural types at the individual level can be quantified on several axes or dimensions, for example boldness, aggression, or exploration [14]. Individuals positioned on the extremes of these axes can be characterized as having different strategies or behavioural types [10], which may affect how individuals perform when facing different situations [15]. A standard test has been developed to measure exploration in a novel environment, scoring the behaviour in a testing room with simulated trees [6, 16]. According to the number of movements in the room, birds are categorized as fast (upper limit) or slow (lower limit) explorers [16]. In addition, birds on the extremes of exploration are considered as proactive if they are fast, or reactive if, on the contrary, they are slow [17]. Given that exploratory behaviour has been measured under standardized conditions and correlates with other behavioural traits, it is considered as a proxy of personality in several bird species and populations, including great tits [8, 16, 18].

There is a growing interest for studying the relationship between consistent between-individual differences in behaviour and cognitive abilities [19]. Although the study of personality has provided insight into behavioural differences between individuals, given the complexity of studying cognitive traits [19] a complete understanding of the relationship between personality and cognitive abilities is still lacking [19, 20]. Habituation is an expression of behavioural plasticity and a basic and widespread form of learning that provides a mechanism to decrease responsiveness to non-threatening situations [21, 22]. Repeated stimulation with harmless cues may help animals to learn what not to fear [23], resulting in a decrease in behavioural responses. Territorial establishment and defence, predation, cuckoldry and food acquisition are some of the inter- and intra-specific challenges that all animals may permanently face. Since behavioural responses to these situations require time and energy, learning to recognize non-threatening signals [19] may prevent unnecessary behavioural responses (fleeing, aggressiveness, escalation), thus providing fitness benefits [23].

Considering the context of birdsong, playback experiments have shown that songbirds may display habituation when responding to a simulated territorial intruder by decreasing their behavioural responses after repeated stimulation with harmless cues, both in the short and long-term [24–26]. Given that this widespread form of learning seems to be a consistent behaviour in songbirds [24], it has been hypothesised that it must provide fitness benefits. Field studies have suggested that signal recognition may help to explain habituation in the short- and long-term. Songbirds may benefit from learning to recognize the song of neighbouring conspecific birds with stable territories that are not a threat, and by, therefore, decreasing behavioural responses, while strongly responding to unknown foreigners that may represent a risk for territory and/or partner tenure [27–29]. However, and despite the fact that habituation in songbirds has been studied for a long time [24], there is no clear understanding of the mechanisms driving the between-individual differences in this basic cognitive trait [19] after repeated stimulation.

In general, between-individual differences in behaviour may show seasonal variation in response to different factors. For instance, it is well known that behavioural responses during the reproductive period may be influenced by reproductive investment [30]. Nest defence is one of the most common behaviours during the reproductive period, and theory predicts that parents may modulate this behaviour in relation to the value of the offspring, which may increase fitness benefits for the parents [30]. A good example is provided by field experiments showing that intensity of nest defence varies between males and females, and also with regard to brood size, sex-ratio and age of the young in the nest [31, 32]. Since behavioural responses seem to be modulated in a complex way, studying and disentangling the role of different factors in the regulation of behaviour may help us to understand variation in behaviour among individuals.

Great tits (*Parus major*) are a very interesting model for studying the mechanisms driving between-individual differences in cognitive abilities. Great tit males are well-known for responding actively to a playback stimulus [33] and they are also known to recognize non-threatening signals by decreasing behavioural responses after repeated stimulation (habituating) both in the short [26, 34] and long-term [25]. Moreover, great tits have been used as a model for the study of personality in songbirds, and standard tests have been developed to measure exploration and personality [6, 16]. Using a suburban great tit population as a model, we aimed to understand the mechanisms underlying between-individual differences in habituation, a proxy of learning speed, in songbirds. Given that previous studies have shown that personality, age, and reproductive investment are related to between-individual differences in behaviour [8, 11, 18], we designed a study to test the role of these factors in the expression of learning speed, as a cognitive trait. Using an experimental approach, we first tested great tit males in a standardized way in the laboratory for exploration in a novel environment, as a proxy of personality, and then, we performed a playback experiment under field conditions creating repeated stimulation to test for habituation and assess learning speed. Finally, we analysed whether the between-individual differences in habituation could be explained by personality, age and/or clutch size, as a proxy of reproductive investment. Formulating a priory expectation for a relationship between personality and cognitive traits is a very difficult task [19]. However, given that slow explorers are known to obtain detailed information from the environment and learn faster [15, 35], we expect that slow explorers habituate faster, showing a more pronounced decrease in behaviour after repeated stimulation than fast explorers.

## Materials and methods

### Ethics statement

The study was performed under proper legislation of the Belgian and Flemish law and was approved by the ethical committee of the University of Antwerp (ID number 2006/22). The Belgian Royal Institute for Natural Sciences (Koninklijk Belgisch Instituut voor Natuurwetenschappen) provided ringing licenses for the authors and technical personnel. The methods that we used (song recording, nest checking, ringing, playback experiment) created only a very low level of stress that did not cause any desertion from nestling activity or mortality.

### Study area and general procedures

Data were collected in 2011 in an established coloured-ringed suburban nest box population of great tits, located on the campus of the University of Antwerp, Wilrijk, Belgium. The population is permanently monitored, with night checks at winter, when birds use nest boxes to sleep, and nest box checks during the day in spring and for part of the summer, following the course of the breeding season. All individuals receive a metal-ring with a unique number when handled as nestlings or when first caught. In addition, all adults receive a combination of three colour-rings that enables individual identification at any time in the field. Permanent monitoring of the population enabled us to assess the exact age of birds that hatched in our population and of immigrants that are first caught as yearlings. Immigrant birds that are first caught as adults are only categorized as adults. In addition, standard morphological measurements (tarsus length, body mass, wing length) are registered every time a bird is caught [36].

### Capturing method and housing

Nest boxes were inspected six times at night during winter (February/March), and only great tit males (adults and yearlings) were captured while sleeping. Individuals were temporarily kept inside darkened wooden boxes and were transported to the laboratory within an hour of being caught. At the laboratory, all individuals were weighed to the nearest 0.1 g using an electronic balance, were housed individually and tested for their exploration behaviour in a novel environment the next morning (see below) [6, 16]. After the test, all individuals were weighed and released at the same place that they were caught.

Following previous studies [6, 16], individuals were housed in standard cages 0.9 x 0.4 x 0.5 m, with a solid bottom and top, rear and sidewalls, a wired-mesh front and three perches. Cages were connected to an observation room through a 20 x 20 cm sliding door. Each cage was previously prepared and provided with sunflowers, water and mealworms. The cages were exposed to natural light and human contact was kept to a minimum before the exploration test.

### Exploration test

A total of 62 individuals were tested for exploration in a novel environment between February and March following a standardized protocol suggested in previous studies [6, 16]. During the test, each male was allowed to enter the observation room (same dimensions as in the previous studies: 4 x 2.4 x 2.3 m), and an observer registered the behaviour for a period of two minutes [6, 16, 18]. Individuals were allowed to enter the room through the sliding doors without handling them. The observer darkened the room before the test, and then, from the inside, slid open the door of the subject male. Subsequently, the observer positioned himself outside the room and turned the light on afterwards, which motivated the bird to enter the observation room. The sliding door was then closed from the outside and the observer could register the exploration test through a polarized window over the two-minute period. The birds were allowed to enter the room in such a way that the process of sliding the doors from the outside kept the human contact to a minimum.

The room contained five artificial wooden trees, each with four short cylindrical branches [6, 16, 37, 38]. During the test, the observer registered both the movements between the artificial trees, sliding doors and lamps (flights) and the short movements between branches of the same tree (hops), but not upon the same branch. The sum of all different movements (flights + hops) over a two-minute period was considered as the measurement of exploratory behaviour [16, 18]. Each great tit male was tested individually in the morning between 09:00 and 11:00. Given that birds experienced natural light conditions, they could eat before the test, and all of them were released before noon at the same place that they were caught. Pearson correlations were used to test for possible effects of possible confounding variables on exploration scores. We tested for the effects of morphology (tarsus length, wing length, body mass), body condition (body mass corrected by tarsus length), age, date of the personality test or average weight loss overnight.

Exploratory behaviour has been shown to be repeatable in most great tit populations [16, 17, 35]. Moreover, repeated tests of the same individuals between 2010 and 2015 in this population have shown that exploration for males is repeatable (repeatability = 0.47 CI = 0.41–0.53)[38], and hence can be considered as a proxy of personality in this population [16].

### Playback experiment

A playback experiment was designed to test for habituation. Daily surveys during pair formation and nest building in the population enabled us to identify the males (and their territories) that were previously tested for exploration in a novel environment. From the individuals tested in the exploration room, a total of 29 males that were not previously exposed to playback experiments (independent of the age) were traced and selected for the playback experiments. These 29 great tit males were recorded at dawn during egg laying, following a standardized recording protocol in order to obtain a reliable estimation of the song repertoire [39]. This was then used to make the playback stimuli. The playback experiments were performed during the incubation period, when females are inside the nest box, and are, therefore, less likely to interfere [34]. During incubation, great tit males regularly visit their female to provide nuptial gifts and stay in the surroundings of the nest box, displaying active territorial defence and response against playbacks [34, 40].

Each great tit male selected for the experiment was exposed three times, with one day in between trials, to a playback consisting of a five-minute looping song. The stimulus was made up of a single two-note song type (see below for details on stimulus) from each bird’s own song repertoire, which has been previously shown to elicit strong behavioural responses and singing behaviour [33, 39]. The same stimulus was used during the three trials. All playbacks were performed in the morning under similar meteorological conditions (no rain, 10–15°C). Following previous studies [34, 39], an Anchor Minivox loudspeaker was placed at an approximated distance of five meters in front of the nest box. This may increase the level of the threat and maximize the response [41]. The speaker was connected through a 20-meter cable to an M-Audio MicroTrack II Professional Mobile Digital Recorder. All playback sessions were recorded from an approximated distance of 20 meters by using a directional microphone (Sennheiser Me67/K6) attached to a portable Marantz PMD660 digital recorder.

Two observers assessed the different behaviours during the playbacks. The first observer (HR) recorded the playback session and while recording continuously registered the approximated distance from the speaker and height from the ground at which the focal male was positioned. Flagging tape placed before the start of the playback at 5 and 10 meters from the speaker in four different directions helped the observer to estimate the distance. This data was used to estimate approach distance, which was considered as the straight line between the bird and the speaker and it was calculated using the Pythagorean theorem. As approach distance was registered continuously during the test, we could determine the distance every time that a bird moved. The second observer (TM) registered the time from the beginning of the test to the first response of the bird (latency) and used a counter to register the number of flights and hopping behaviour (small jumps on a branch). The sum of flights and hops was considered as a proxy of locomotor behaviour. A pair-wise Pearson correlation was performed between behavioural responses. Given that approach distances were correlated, we decided to include only the closest approach distance. We first placed the loudspeaker and the flagging tape to help estimate the distance from the speaker, and after confirming the presence of the focal male we waited for five minutes to enable the bird to acclimatize to the experimental conditions before starting the playback. All playback data and correlations between behavioural responses are available as supplementary material (S1 and S2 Tables).

From the recordings made during the experiment, we obtained information on the number of strophes sung as a response to the playback and the number of strophes overlapping the playback. We considered that a strophe sung by the focal male overlapped the playback only when the bird started singing even though there was a strophe playing from the playback [42]. From the behavioural observations we obtained information on latency, locomotor behaviour (number of hops + flights) during playback, closest approach distance (minimum straight distance from the speaker) and average approach distance to the speaker (sum of the registered distances/number of times a different distance was registered).

### Playback stimuli

The stimuli were created using Avisoft-SAS Lab Pro software version 5.0 Germany (Avisoft Bioacoustics, Berlin, Germany). From the recordings made at dawn during the egg-laying period in 2011, we selected a simple two-notes song type from each subject male. Based on the quality of the recording (signal-to-noise ratio), a strophe of approximately 3 s per song type was selected. The selected strophes were trimmed, filtered at 1500 Hz, and their amplitude was normalized at 75% of a volt. A silent gap of 3 s was inserted after each strophe, and both strophe and gap were copied and appended several times in order to create a five-minute lopping song.

### Statistical analysis

Data were tested for normality using a Kolmogorov-Smirnov test and homogeneity of variance was tested using Levene’s test. As data fulfilled assumptions for normality and homoscedasticity, we used parametrical tests when necessary. Two different tests were performed. First, we analysed the song and behavioural responses of the males during the three playbacks. We used a linear mixed-effect model (LMM), which enables the analysis of repeated measurements to assess within-individual variation, while controlling for non-independence of repeated data [43]. In the model, individuals were set as random factor; repeat was set as fixed factor and behavioural responses (strophes, overlapping, latency, locomotor behaviour (number of movements: hops and flights) and closest approach distance to the speaker) as response variables. We included age as a fixed factor in the analysis to test for possible effects of accumulated experience. Age was considered as a continuous variable between 1 and 4 years of age (N for ages 1 to 4: 15, 9, 4, 1). This test aimed to investigate whether behavioural (latency, locomotor behaviour, closest approach distance) and song responses (strophes sung as response during playback and overlapping) decreased with the presentation of repeated stimuli.

Given that some behavioural responses significantly decreased with repeated stimulation (see below in results), the second test aimed to investigate whether the decrease in response was predicted by clutch size, exploratory behaviour and/or age. Using the data of behavioural and song responses that significantly decreased during the three playback experiments, we fitted linear mixed-effect models (LMM) per response variable. In each model, we set one of the behavioural responses (overlapping, locomotor behaviour, closest approach distance to the speaker) as response variable, individuals were set as subjects and order of playback was used as repeat. Exploration score, clutch size and age (continuous) were used as factors. Given that this test attempted to see whether the exploration and reproductive investment could predict the decrease in response, we included the one-way interactions between these variables and repeat as factors. Using different combinations of factors, we set a total of 16 different models per response variable, and the best model was chosen based on the lowest Akaike information criterion factor (AICc). All analyses and figures were performed in JMP Pro 12.0 for Mac (SAS Institute Inc. NC, USA). Descriptive statistics are presented as average ± S.E. unless otherwise stated.

## Results

### Exploration in a novel environment

Exploratory behaviour ranged between 0 and 41 movements (22.65 ± 10.51, *N* = 29) (S3 Table). Exploration was not related to morphology (tarsus length, wing length, body mass), body condition (body mass corrected by tarsus length) or age (all *P*>0.7 *N* = 29). Moreover, exploratory behaviour was not related to the date of the personality test (r = 0.26, *P* = 0.16, *N* = 29). Average weight loss overnight was within the natural limits for the species (1.06 ± 0.6 g) [44] and was not related to exploratory behaviour (r = -0.16 *P* = 0.41 *N* = 29).

### Habituation

**Fig 1:**
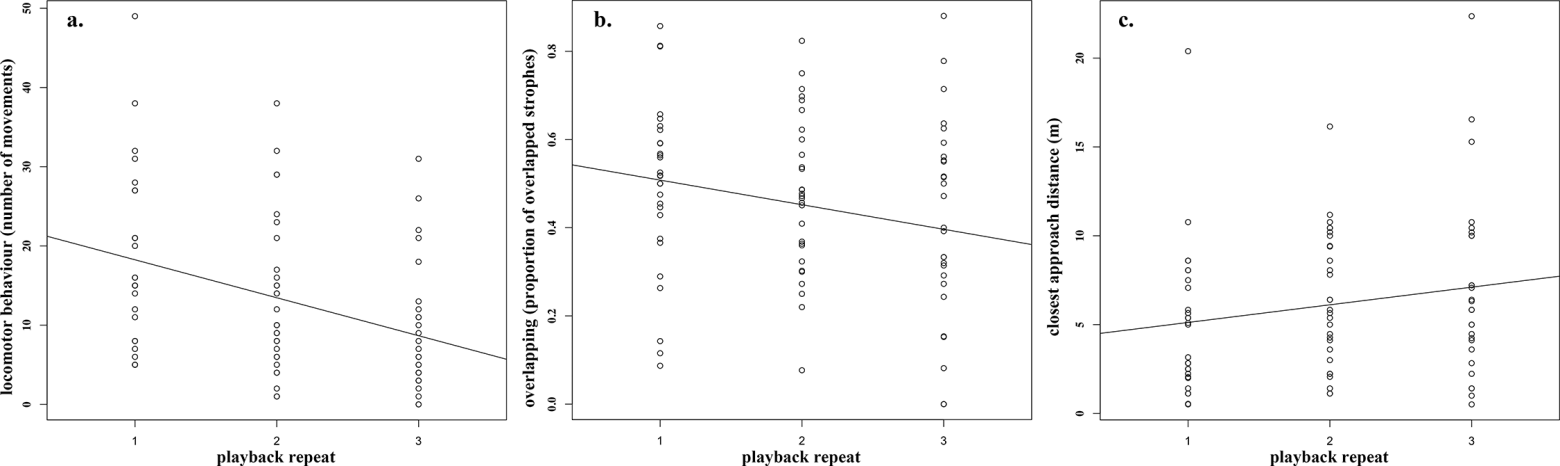
Decrease of behavioural responses with repeated stimulation for a. locomotor behaviour expressed as the number of flights and hops around the speaker (R^2^ = 0.15); b. closest approach distance (R^2^ = 0.03), and c. song overlapping (R^2^ = 0.05).

Behavioural response of great tit males significantly decreased after repeated stimulation (Table 1, Fig 1). LMM indicated that male great tits significantly moved less during playback, stayed further away and overlapped less with successive playback stimulation (Table 1). On the other hand, latency and the number of strophes sung as response to the playback did not change with repeated stimulation (Table 1), similar to a previous study [34]. Age did not have a significant effect on the model (Table 1).

**Table 1:** Linear mixed effect model of behavioural responses during playbacks.

Variables	Age					Repeat				
	Estimate	S.E.	DF	tratio	Probability	Estimate	S.E.	DF	tratio	Probability
Latency	-1.217	5.106	27	-0.24	0.814	-3.578	4.277	57	-0.84	0.406
Locomotor behaviour	2.844	1.742	27	1.63	0.114	**-4.793**	**0.819**	**57**	**-5.85**	**< .0001**
Closest approach distance	-0.538	0.817	27.17	-0.66	0.516	**1.080**	**0.391**	**55.5**	**2.76**	**0.008**
Strophes sung	-4.199	2.473	27	-1.7	0.101	-2.603	1.739	57	-1.5	0.140
Overlapping	-0.033	0.031	27	-1.06	0.298	**-0.056**	**0.024**	**57**	**-2.32**	**0.024**

Text in bold represents significant values.

### Habituation, exploration and clutch size

**Fig 2:**
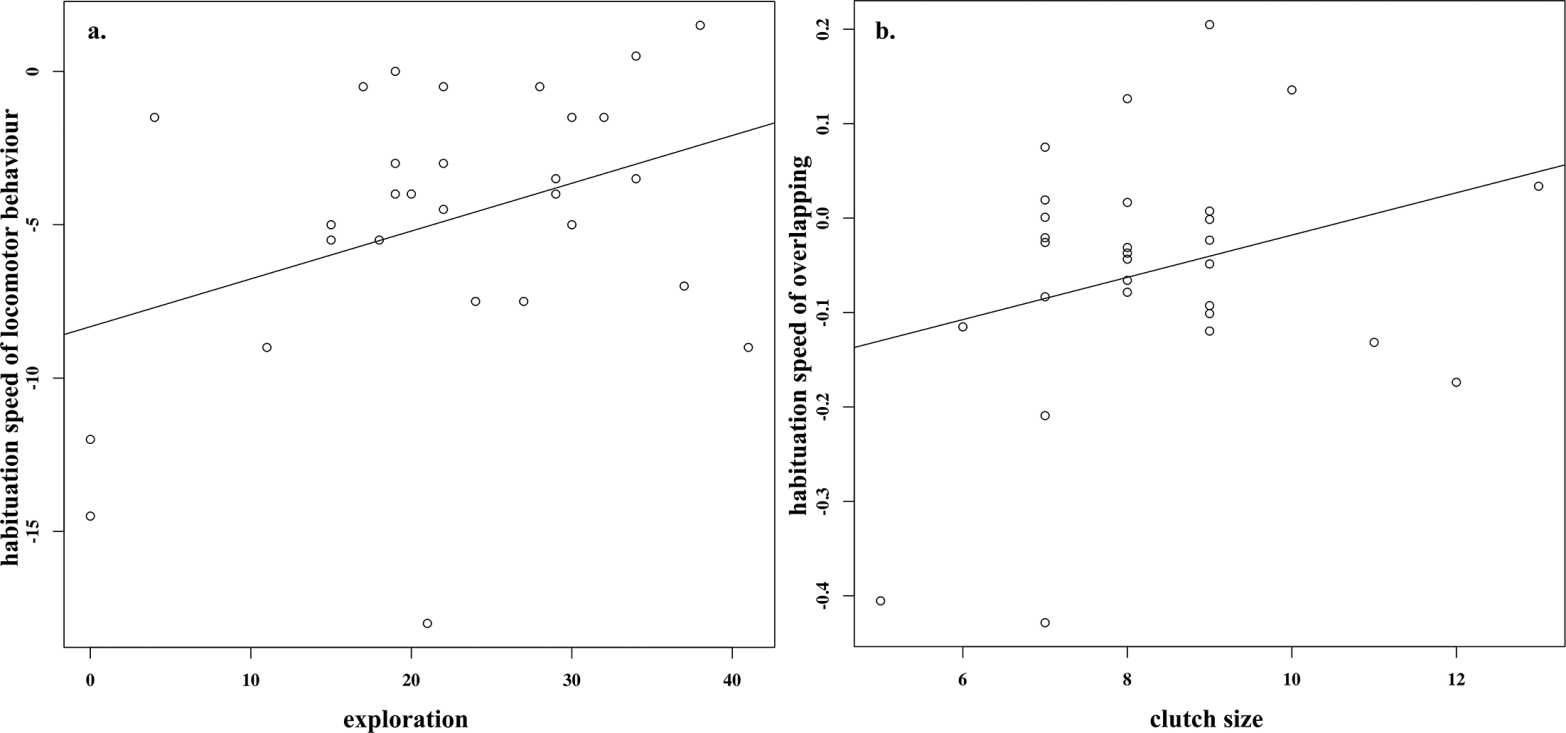
Relationship between slope of behavioural responses (habituation speed) to the playback and factors for: a. Locomotor behaviour (number of flights and hops around the speaker) and exploration in a novel environment; and b. Overlapping (proportion of overlapping strophes) and clutch size. Slopes were calculated using linear regression models between playback trials and behavioural responses. Slopes are a proxy of habituation speed, showing how fast an individual decreases its response with repeated stimulation. Habituation speed is measured from fast to slow, resulting in negative slope values for fast decrease in response; slope values close to zero for slow or no habituation; and an increase in behavioural response is represented by slope values above zero.

Results of LMM indicated that habituation of locomotor behaviour and song overlapping was explained by exploration and clutch size (Table 2). The best model for locomotor behaviour included repeat and its interaction with exploration (S4 Table). Fast explorers habituated less or did not habituate (Fig 2A). On the other hand, between-individual differences in habituation of song overlapping were significantly predicted by repeat and its one-way interactions with exploration and clutch size (Table 2). While fast explorers habituated more, individuals with a larger clutch size showed less habituation (Fig 2B). Finally, habituation of minimum approach distance was not significantly predicted by exploratory behaviour, clutch size or age (Table 2).

**Table 2:** Best linear mixed effect models explaining behavioural responses that habituated.

Behaviours	Factor	Estimate	S.E.	DFDen	t Ratio	P	95% L	95% U
Locomotor behaviour	Exploration*Repeat	**0.159**	**0.075**	**27**	**2.11**	**0.044**	**0.004**	**0.313**
Minimum distance	Exploration*Repeat	-0.041	0.032	25.8	-1.25	0.221	-0.107	0.026
Overlapping	Exploration*Repeat	**-0.005**	**0.002**	**26**	**-2.29**	**0.030**	**-0.010**	**-0.001**
	Clutch Size*Repeat	**0.037**	**0.014**	**26**	**2.59**	**0.016**	**0.008**	**0.067**

Text in bold represents significant values. Repeat was included in all models, but it is not shown in this table, because Table 1 shows similar information. Factors represent interaction between variables.

## Discussion

To try to understand the mechanisms affecting between-individual differences in a basic cognitive trait (habituation), we designed an experimental approach, first testing exploration behaviour in a novel environment, as a measurement of personality, and second, performing a series of playback experiments in the field creating a repeated stimulation for analysing the decrease in behavioural responses in relation to age, reproductive investment and personality. Similar to previous studies [25, 26, 34], our results revealed that great tit males learnt to recognize the experimental setup of a playback experiment and decreased their response after repeated stimulation. Birds decreased locomotor behaviour, expressed as the numbers of hops and flights, and also stayed further away every time that a new playback was performed, indicating a decrease in aggressiveness when confronted repeatedly with the same stimulus [25]. This suggests that birds may learn to recognize the experimental setup as a non-threatening event [25]. Song overlapping also showed a pattern of decreased response, while latency and the number of strophes sung as response did not show a consistent pattern of change, similar to previous studies [25, 34]. Our study also revealed that the degree to which birds decrease their behaviour (how fast they learn) is significantly related to exploration behaviour (personality) and reproductive investment.

We showed that between-individual differences in habituation towards a playback stimulus, an indirect measurement of learning speed, are affected both by exploration behaviour and reproductive investment. Given that different behaviours are expressed during a behavioural interaction (i.e. territorial challenge), the rate of habituation of the different behaviours may be different [24, 25], and each behaviour may be affected by a different factor, which seems to be the case in our study.

Habituation, understood as a basic cognitive process, requires that individuals pay attention to the cues, and collect information to achieve recognition of a non-threatening situation. Theory predicts that personality may affect how individuals collect information from the environment [11]. Individuals in the extremes of a behavioural type (as shown by exploration in a novel environment), may show opposite strategies, and then, this may influence how much information they get from the environment, and may, therefore, affect the expression of cognitive abilities. In great tits, fast explorers (also termed proactive copers, see Carere et al. [17]) are individuals that react faster, are more aggressive, are bolder, make rapid decisions, are relatively insensitive to external stimuli, make routines, and show increased levels of testosterone [2, 10, 17, 45, 46]. On the other hand, slow explorers (or reactive individuals [17]) are more cautious when making decisions, they are more sensitive to external stimulation, are shyer and they have relatively low levels of aggressiveness [2, 10, 45, 46]. Therefore, fast explorer birds may collect less information from the environment during a behavioural interaction, impairing their ability to make decisions, which may limit the capacity of displaying cognitive abilities, as in the case of recognition of non-threatening signals or habituation.

A decrease in behavioural responses after repeated stimulation (habituation) is a plastic behaviour, which is the result of a basic learning or recognition process that animals make after repeated stimulation with harmless cues [24]. Our results strongly suggest that fast explorers collected less information during territorial interactions, remaining aggressive (remained moving) after three playbacks, displaying less behavioural plasticity than slow explorers and showing in consequence a delay in learning speed. Our results concur with theoretical predictions [19] and previous studies where fast-exploring great tits collected less information while visiting feeders in the wild [35], similarly to fast-exploring black capped chickadees (*Poecile atricapillus*) that took longer to learn a task [15]. When birds are repeatedly confronted with non-threatening situations, they need to recognize that a particular signal does not imply a threat. During the recognition process the individuals need to collect information using different sensorial modalities, in order to make a basic cognitive process. Reactive birds, take a longer time to make decisions [17] and shorter time to learn tasks [15], which seems to suggests they may collect more information from the environment [35], enabling them to decrease their behavioural response quickly after repeated stimulation, showing behavioural plasticity, and avoiding unnecessary responses. Alternatively, proactive birds remain aggressive, and they are unable to change their behaviour.

Our study also revealed that reproductive investment might also play a role in modulating behavioural plasticity during behavioural interactions. Male great tits paired to females with larger clutches remained overlapping after repeated stimulation and did not habituate. Although there is still debate, song overlapping is considered as a signal of aggressive intent [42]. In agreement with theoretical predictions and previous studies, the value of the offspring determined the behavioural response of males [30–32]. Losing a larger clutch may imply a higher cost for males, which may, therefore, motivate them to display active nest defence. Previous studies have also found that song behaviour remains constant with repeated stimulation [25, 34, 47], which may indicate that locomotor behaviour and song response provide different information during territorial interactions. On the other hand, habituation of song overlapping also seems to be affected by personality, but in the opposite way of locomotor behaviour.

Finally, the habituation on closest approach distances was not explained by the factors we were testing. It is possible that habituation in approaching distance is modulated by a factor that we did not consider in this study. This finding also supports our idea that the different components of behavioural responses provide different information, and that each behaviour may be affected by a different factor.

Between individual differences in a basic cognitive trait such as habituation are modulated in a complex way, and both ultimate and proximate factors may play a role in the expression of behaviour. One form of behavioural plasticity, such as the decrease in behavioural responses after repeated stimulation (habituation), may be simultaneously modulated by personality and reproductive investment. Given that during a behavioural interaction different behaviours may be involved, different neuroendocrine mechanisms may regulate each behaviour [24], affecting the gathering of information and the recognition of signals, therefore driving the rate of habituation of each behaviour. We experimentally demonstrated that a particular personality type affects the rate of habituation of locomotor behaviour, while the rate of decrease in overlapping was simultaneously modulated by reproductive investment and personality. It is important to disentangle the role of multiple factors in the regulation of behaviour to acquire a better understanding of the proximate and ultimate factors that modulate cognitive abilities.

## Supporting information

S1 Table Behavioural responses during playback experiment. (PDF)

S2 Table Pearson pair-wise correlation between behavioural responses. Text in bold represents significant values.
(PDF)

S3 Table Exploration score and Clutch size for individuals tested during playback experiment. (PDF)

S4 Table Results for all linear mixed effect models run during statistical analysis. (PDF)
