# Supplementary material for: Learning speed is affected by personality and reproductive investment in a songbird
Source: PLoS One. 2017 Oct 11;12(10):e0185410. doi: 10.1371/journal.pone.0185410 (PMC5636094; doi:10.1371/journal.pone.0185410)
Supplement: S1 Table — (PDF) [file pone.0185410.s001.pdf]

ring	repeat	date	latency	totalmov	avedist	mindist	strophes	overlapping
52v47648	1	14-Apr-11	84.97	38	2.93	0.54	24	0.375
52v47648	2	16-Apr-11	3.51	24	5.9	4.27	54	0.537
52v47648	3	18-Apr-11	13.41	2	10.19	10.19	33	0.3333
52v47669	1	14-Apr-11	20.62	16	6.88	5.09	34	0.6471
52v47669	2	16-Apr-11	0	5	10.18	5.83	39	0.6667
52v47669	3	18-Apr-11	4.51	9	16.82	10.77	20	0.55
52v47668	1	25-Apr-11	9.43	15	6.03	5.38	65	0.4462
52v47668	2	27-Apr-11	7.52	4	10.2	10.2	43	0.3023
52v47668	3	29-Apr-11	3.17	5	9.98	5.83	35	0.3143
52v47265	1	13-Apr-11	9.07	15	9.19	7.5	46	0.6304
52v47265	2	15-Apr-11	4.12	10	8.69	5.65	43	0.6977
52v47265	3	17-Apr-11	8.43	8	7.77	2.83	35	0.4
52v47680	1	22-Apr-11	8.16	5	8.63	5.65	37	0.6216
52v47680	2	24-Apr-11	19.15	14	8.92	5	37	0.4865
52v47680	3	26-Apr-11	14.66	5	8.36	5.83	11	0.6364
51v19961	1	19-Apr-11	19.48	11	12.06	8.06	7	0.8571
51v19961	2	21-Apr-11	81.1	4	13.58	10.77	21	0.7143
51v19961	3	23-Apr-11	46.25	3			0	0
50v03197	1	23-Apr-11	2.37	7	10.44	8.06	50	0.5
50v03197	2	25-Apr-11	3.08	6	10.6	10.44	50	0.22
50v03197	3	27-Apr-11	3.09	1	10.44	10.44	46	0.1522
52v47630	1	27-Apr-11	187.3	16	3.76	2	33	0.4545
52v47630	2	29-Apr-11	33.1	23	4.88	3	71	0.4507
52v47630	3	1-May-11	4.18	13	10.85	6.32	51	0.3922
52v47317	1	13-Apr-11	119.19	12	5.45	2.83	27	0.5185
52v47317	2	15-Apr-11	5.56	14	6.78	2.23	53	0.4717
52v47317	3	17-Apr-11	5.56	6	6.78	2.23	53	0.4717
49v23589	1	21-Apr-11	16.06	21	6.84	2.83	16	0.8125
49v23589	2	23-Apr-11	4.14	2	8.06	8.06	44	0.25
49v23589	3	25-Apr-11	9.64	6	6.05	3.6	25	0.88
49v23554	1	24-Apr-11	14.99	21	3.6	2.5	19	0.2632
49v23554	2	26-Apr-11	9.63	32	3.79	2.06	10	0.3
49v23554	3	28-Apr-11	10.73	7	7.51	5	36	0
52v47651	1	13-Apr-11	1.85	32	5.87	3.16	40	0.525
52v47651	2	15-Apr-11	9.23	16	7.37	6.4	20	0.75
52v47651	3	17-Apr-11	8.26	3	7.21	7.21	9	0.7778
51v19987	1	20-Apr-11	2.13	15	9.2	8.6	41	0.365853659
51v19987	2	22-Apr-11	2.19	1	16.15	16.15	45	0.6889
51v19987	3	24-Apr-11	9.89	4	11.17	10	24	0.2917
47v94624	1	19-Apr-11	38.2	28	3.02	0.51	22	0.5909
47v94624	2	21-Apr-11	8.03	12	3.47	1.12	34	0.8235
47v94624	3	23-Apr-11	49.64	10	5.09	1.41	27	0.5926
50v03297	1	15-Apr-11	0.71	31	3.18	1.12	35	0.6571
50v03297	2	17-Apr-11	27.86	29	3.8	1.41	35	0.4857
50v03297	3	19-Apr-11	2.41	22	3.88	1	32	0.5

ring	repeat	date	latency	totalmov	avedist	mindist	strophes	overlapping
50v03305	1	25-Apr-11	16.17	14	4.87	1.41	38	0.2895
50v03305	2	27-Apr-11	3.18	15	4.89	3.6	46	0.4565
50v03305	3	29-Apr-11	14.69	11	7.31	4.47	41	0.561
51v19386	1	20-Apr-11	2.8	15	11.44	5	37	0.5676
51v19386	2	22-Apr-11	3.65	9	16.82	8.6	40	0.6
51v19386	3	24-Apr-11	3.91	4	10	10	31	0.5161
52v47244	1	25-Apr-11	17.4	8	7.69	5.83	23	0.087
52v47244	2	27-Apr-11	22.6	1	11.18	11.18	15	0.4667
52v47244	3	29-Apr-11	3.57	0			1	0
49v24798	1	14-Apr-11	47.72	8	25.72	20.39	22	0.5
49v24798	2	16-Apr-11	7.74	15	9.54	6.4	38	0.3684
49v24798	3	18-Apr-11	178.88	0	22.36	22.36	3	0.3333
52v47532	1	16-Apr-11	79.4	21	11.55	5.65	37	0.8108
52v47532	2	18-Apr-11	20.9	8	5.38	9.39	46	0.5652
52v47532	3	20-Apr-11	131.1	11	3.62	1.41	0	0
52v47508	1	20-Apr-11	4.14	5	10.52	8.6	64	0.5
52v47508	2	22-Apr-11	4.11	9	10.69	9.43	30	0.5333
52v47508	3	24-Apr-11	2.08	4	15.29	15.29	49	0.0816
52v47657	1	14-Apr-11	47.08	49	5.65	2.05	7	0.4286
52v47657	2	16-Apr-11	1.69	38	5.38	4.12	13	0.0769
52v47657	3	18-Apr-11	5.76	31	9.76	4.12	37	0.2432
52v47637	1	22-Apr-11	4.19	15	7.43	5.38	29	0.5172
52v47637	2	24-Apr-11	0	6	12.98	10.44	65	0.4769
52v47637	3	26-Apr-11	21.68	18	7.2	4.24	35	0.5143
52v47190	1	19-Apr-11	2.69	27	8.75	7.07	49	0.5918
52v47190	2	21-Apr-11	3.43	21	13.27	7.81	44	0.4091
52v47190	3	23-Apr-11	3.61	26	22.41	16.55	48	0.625
49v23865	1	25-Apr-11	6.95	20	3.04	2	59	0.5593
49v23865	2	27-Apr-11	10.67	17	3.01	1.12	50	0.36
49v23865	3	29-Apr-11	32.72	21	1.82	0.51	50	0.32
52v47011	1	24-Apr-11	15.67	6	2.98	2.5	52	0.1154
52v47011	2	26-Apr-11	6.54	7	10.19	4.47	34	0.3235
52v47011	3	28-Apr-11	10.61	3	9.31	7.21	39	0.1538
50v00943	1	13-Apr-11	2.04	27	3.99	1.12	39	0.5641
50v00943	2	15-Apr-11	17.61	29	4.43	2.23	11	0.3636
50v00943	3	17-Apr-11	13.05	3	5.82	5	42	0.7143
52v47261	1	19-Apr-11	10.92	5	13.03	10.77	40	0.475
52v47261	2	21-Apr-11	9.7	10	10.38	10	44	0.2727
52v47261	3	23-Apr-11	5.29	4	7.07	7.07	11	0.2727
51v20508	1	19-Apr-11	39.97	27	4.44	2.23	21	0.1429
51v20508	2	21-Apr-11	0	8	7.63	5.38	45	0.6222
51v20508	3	23-Apr-11	3.37	12	10.97	6.4	38	0.5526

ring	repeat	date	latency	totalmov	avedist	mindist	strophes	overlapping

Columns legend

**ring**	Ring number
**repeat**	Trial number
**date**	Date of test
**latency**	Time from the begining of the tes until first response
**totalmov**	Number of total movements during the test
**avedist**	Average distance to the speaker
**mindist**	Minimum distance to the speaker
**strophes**	Number of strophes sung during playback
**overlapping**	Number of overlapping strophes
