# Supplementary material for: Learning speed is affected by personality and reproductive investment in a songbird
Source: PLoS One. 2017 Oct 11;12(10):e0185410. doi: 10.1371/journal.pone.0185410 (PMC5636094; doi:10.1371/journal.pone.0185410)
Supplement: S2 Table — Text in bold represents significant values. (PDF) [file pone.0185410.s002.pdf]

Learning speed is affected by personality and reproductive investment in a songbird
Hector Fabio Rivera-Gutierrez, Tine Martens, Rianne Pinxten and Marcel Eens

Pearson correlation between behavioral responses

Variable	by Variable	Correlation	Lower 95%	Upper 95%	Signif Prob
**Average distance**	**Locomotor behaviour**	**-0.4117**	**-0.5744**	**-0.2177**	**<.0001**
**Minimum distance**	**Locomotor behaviour**	**-0.5199**	**-0.6599**	**-0.345**	**<.0001**
**Minimum distance**	**Average distance**	**0.9047**	**0.8568**	**0.9371**	**<.0001**
Strophes sung	Locomotor behaviour	-0.0513	-0.2592	0.1611	0.6368
Strophes sung	Average distance	-0.0082	-0.2209	0.2053	0.9408
Strophes sung	Minimum distance	0.0024	-0.2109	0.2154	0.9828
Overlapping	Locomotor behaviour	0.0609	-0.1517	0.2681	0.5751
Overlapping	Average distance	0.0863	-0.1292	0.2941	0.432
Overlapping	Minimum distancet	-0.0105	-0.2231	0.2031	0.924
Overlapping	Strophes sung	0.1087	-0.1043	0.3122	0.3162
Latency	Locomotor behaviour	0.0443	-0.1679	0.2526	0.6839
Latency	Average distance	0.0115	-0.2021	0.2241	0.917
Latency	Minimum distance	0.0297	-0.1846	0.2413	0.787
**Latency**	**Strophes sung**	**-0.3594**	**-0.5299**	**-0.1609**	**0.0006**
Latency	Overlapping	-0.0718	-0.2782	0.141	0.5089
