# Supplementary material for: Learning speed is affected by personality and reproductive investment in a songbird
Source: PLoS One. 2017 Oct 11;12(10):e0185410. doi: 10.1371/journal.pone.0185410 (PMC5636094; doi:10.1371/journal.pone.0185410)
Supplement: S3 Table — (PDF) [file pone.0185410.s003.pdf]

ring	age	clutchsize	weight_night	tarsus	weight_morning	exploration
52v47648	1	7	17.5	19.88	16.5	21
52v47669	1	9	18.4	20.94	17.5	29
52v47668	1	8	18.8	20.86	18	15
52v47265	1	6	18.2	20.62	17.5	34
52v47680	1	9	20.4	20.38	17	19
51v19961	3	7	18.1	20.59	17	29
50v03197	2	12	17.4	19.38	16.75	19
52v47630	1	8	19.2	19.38	18.5	32
52v47317	1	9	18.3	19.87	18.3	22
49v23589	2	13	19.3	20.84	19	24
49v23554	4	11	18.1	20.36	17	37
52v47651	1	8	19.1	19.96	17.5	0
51v19987	3	8	17.3	19.52	16	18
47v94624	2	7	19.4	20.05	18.75	41
50v03297	2	8	19.2	20.08	18	22
50v03305	2	10	18.6	18.81	17.75	30
51v19386	2	7	17.7	20.02	17.25	15
52v47244	1	8	19	20.36	17	19
49v24798	2	7	17.9	20.62	17.5	20
52v47532	1	5	18.9	19.54	17	30
52v47508	1	7	17.4	19.51	16	28
52v47657	3	9	18.9	20.28	17.5	11
52v47637	1	9	17.7	19.67	17	38
52v47190	1	8	18.3	19.47	17.5	22
49v23865	3	9	19.5	19.42	18	34
52v47011	1	7	18.4	18.85	17.25	4
50v00943	2	7	20	19.91	19	0
52v47261	1	9	19	21.53	18	17
51v20508	2	9	17.6	20.55	16.75	27

**ring**	Ring number
**age**	Exact age in years
**clutchsize**	Number of eggs laid by the female
**weight_night**	Weight at night
**tarsus**	Tarsus lenght in mm
**weight_morning**	Weight in the morning after the test and before being released
**exploration**	Exploration score
