# Supplementary material for: Learning speed is affected by personality and reproductive investment in a songbird
Source: PLoS One. 2017 Oct 11;12(10):e0185410. doi: 10.1371/journal.pone.0185410 (PMC5636094; doi:10.1371/journal.pone.0185410)
Supplement: S4 Table — (PDF) [file pone.0185410.s004.pdf]

Learning speed is affected by personality and reproductive investment in a songbird

Hector Fabio Rivera-Gutierrez, Tine Martens, Rianne Pinxten and Marcel Eens

Results of all GLM

Overlapping Fit Group

Fit Mixed

Actual by Predicted Plot

Fit Statistics

-2 Residual Log Likelihood	1.6010023
-2 Log Likelihood	-48.98695
AICc	-18.00065
BIC	9.0698573

Repeated Effects Covariance Parameter Estimates
Repeated Effect: repeat2

Covariance Parameter	Estimate	Std Error	95% Lower	95% Upper
Var(1)	0.043381	0.0129568	0.0179862	0.0687759
Cov(2,1)	0.0008064	0.0065501	-0.012031	0.0136443
Var(2)	0.0239166	0.0071038	0.0099934	0.0378398
Cov(3,1)	0.0209085	0.0118868	-0.002389	0.0442063
Cov(3,2)	0.0131193	0.0082588	-0.003068	0.0293062
Var(3)	0.0623638	0.018777	0.0255617	0.099166

Fixed Effects Parameter Estimates

Term	Estimate	Std Error	DFDen	t Ratio	Prob>|t|	95% Lower	95% Upper
Intercept	0.7637843	0.1460161	30.4	5.23	<.0001	0.4657595	1.0618091
repeat	-0.056738	0.0246377	26	-2.3	0.0295	-0.107381	-0.006094
age	-0.009034	0.0324675	25	-0.28	0.7831	-0.075902	0.0578346
clutchsize	-0.030141	0.0164979	25.3	-1.83	0.0795	-0.064099	0.003816
total_mov	0.0034197	0.0025564	24.9	1.34	0.1931	-0.001846	0.0086853
(total_mov-22.6552)*(repeat-2)	-0.004196	0.0023952	26	-1.75	0.0916	-0.009119	0.0007276
(clutchsize-8.31034)*(repeat-2)	0.0266311	0.0148732	26	1.79	0.085	-0.003941	0.0572034

Fixed Effects Tests

Source	Nparm	DFNum	DFDen	F Ratio	Prob > F
repeat	1	1	26	5.3032862	0.0295
age	1	1	25	0.0774144	0.7831
clutchsize	1	1	25.3	3.3378377	0.0795
total_mov	1	1	24.9	1.7895564	0.1931
total_mov*repeat	1	1	26	3.0687235	0.0916
clutchsize*repeat	1	1	26	3.2060663	0.085

Fit Mixed

Actual by Predicted Plot

Fit Statistics

-2 Residual Log Likelihood	-3.522591
-2 Log Likelihood	-49.02738
AICc	-20.81116
BIC	4.5635191

Repeated Effects Covariance Parameter Estimates
Repeated Effect: repeat2

Covariance Parameter	Estimate	Std Error	95% Lower	95% Upper
Var(1)	0.042872	0.0127678	0.0178476	0.0678964
Cov(2,1)	0.0002664	0.0063866	-0.012251	0.0127839
Var(2)	0.0233456	0.0068791	0.0098628	0.0368284
Cov(3,1)	0.0208824	0.0116874	-0.002025	0.0437893
Cov(3,2)	0.0130622	0.0079677	-0.002554	0.0286785
Var(3)	0.0628205	0.0185724	0.0264193	0.0992217

Fixed Effects Parameter Estimates

Term	Estimate	Std Error	DFDen	t Ratio	Prob>|t|	95% Lower	95% Upper
Intercept	0.7649269	0.144217	31.8	5.3	<.0001	0.4710986	1.0587553
repeat	-0.056738	0.0246377	26	-2.3	0.0295	-0.107381	-0.006094
clutchsize	-0.03192	0.0155441	26	-2.05	0.0502	-0.063871	3.16E-05
total_mov	0.0033563	0.0025033	26	1.34	0.1916	-0.001789	0.0085019
(total_mov-22.6552)*(repeat-2)	-0.004196	0.0023952	26	-1.75	0.0916	-0.009119	0.0007276
(clutchsize-8.31034)*(repeat-2)	0.0266311	0.0148732	26	1.79	0.085	-0.003941	0.0572034

Fixed Effects Tests

Source	Nparm	DFNum	DFDen	F Ratio	Prob > F
repeat	1	1	26	5.3032858	0.0295
clutchsize	1	1	26	4.2168576	0.0502
total_mov	1	1	26	1.7976341	0.1916
total_mov*repeat	1	1	26	3.0687231	0.0916
clutchsize*repeat	1	1	26	3.2060656	0.085

Fit Mixed

Actual by Predicted Plot

Fit Statistics

-2 Residual Log Likelihood	-7.714757
-2 Log Likelihood	-46.43168
AICc	-20.91168
BIC	2.6933073

Repeated Effects Covariance Parameter Estimates
Repeated Effect: repeat2

Covariance Parameter	Estimate	Std Error	95% Lower	95% Upper
Var(1)	0.0389553	0.010973	0.0174486	0.0604619
Cov(2,1)	0.0024318	0.0057471	-0.008832	0.0136959
Var(2)	0.0221531	0.0062482	0.0099068	0.0343994
Cov(3,1)	0.0224357	0.0124106	-0.001889	0.04676
Cov(3,2)	0.0124414	0.0087976	-0.004802	0.0296843
Var(3)	0.0745791	0.0217308	0.0319875	0.1171708

Fixed Effects Parameter Estimates

Term	Estimate	Std Error	DFDen	t Ratio	Prob>|t|	95% Lower	95% Upper
Intercept	0.8330652	0.1412819	32.5	5.9	<.0001	0.5454483	1.1206821
repeat	-0.053054	0.02498	27	-2.12	0.043	-0.104309	-0.001799
clutchsize	-0.04177	0.0150819	26	-2.77	0.0102	-0.072772	-0.010769
total_mov	0.003697	0.0025099	25.7	1.47	0.1529	-0.001465	0.0088589
(total_mov-22.6552)*(repeat-2)	-0.003275	0.002419	27	-1.35	0.187	-0.008238	0.0016885

Fixed Effects Tests

Source	Nparm	DFNum	DFDen	F Ratio	Prob > F

repeat	1	1	27	4.5107735	0.043
clutchsize	1	1	26	7.6705655	0.0102
total_mov	1	1	25.7	2.1696228	0.1529
total_mov*repeat	1	1	27	1.8327935	0.187

Fit Mixed

Actual by Predicted Plot

Fit Statistics

-2 Residual Log Likelihood	-10.87986
-2 Log Likelihood	-45.90856
AICc	-20.38856
BIC	3.216428

Repeated Effects Covariance Parameter Estimates
Repeated Effect: repeat2

Covariance Parameter	Estimate	Std Error	95% Lower	95% Upper
Var(1)	0.0405086	0.011751	0.017477	0.0635401
Cov(2,1)	0.0015665	0.0060423	-0.010276	0.0134093
Var(2)	0.0226337	0.0065043	0.0098855	0.035382
Cov(3,1)	0.0214785	0.0122154	-0.002463	0.0454202
Cov(3,2)	0.0129021	0.0086249	-0.004002	0.0298066
Var(3)	0.0713994	0.0213203	0.0296124	0.1131864

Fixed Effects Parameter Estimates

Term	Estimate	Std Error	DFDen	t Ratio	Prob>|t|	95% Lower	95% Upper
Intercept	0.74751	0.1446947	32.5	5.17	<.0001	0.4529686	1.0420514
repeat	-0.054334	0.0254321	27	-2.14	0.0419	-0.106517	-0.002152
clutchsize	-0.034527	0.0156112	25.6	-2.21	0.0361	-0.066639	-0.002414
total_mov	0.0049084	0.0024287	26	2.02	0.0537	-8.38E-05	0.0099006
(clutchsize-8.31034)*(repeat-2)	0.0195838	0.0152924	27	1.28	0.2112	-0.011794	0.0509612

Fixed Effects Tests

Source	Nparm	DFNum	DFDen	F Ratio	Prob > F
repeat	1	1	27	4.5644385	0.0419
clutchsize	1	1	25.6	4.8913896	0.0361
total_mov	1	1	26	4.0844884	0.0537
clutchsize*repeat	1	1	27	1.6400097	0.2112

Fit Mixed

Actual by Predicted Plot

Fit Statistics

-2 Residual Log Likelihood	-17.49567
-2 Log Likelihood	-41.94441
AICc	-19.04967
BIC	2.7146734

Repeated Effects Covariance Parameter Estimates
Repeated Effect: repeat2

Covariance Parameter	Estimate	Std Error	95% Lower	95% Upper
Var(1)	0.0403182	0.0115708	0.0176398	0.0629966
Cov(2,1)	0.0031486	0.0063494	-0.009296	0.0155931
Var(2)	0.0259882	0.0074441	0.0113981	0.0405783
Cov(3,1)	0.0167249	0.0106203	-0.004091	0.0375403
Cov(3,2)	0.009921	0.0080023	-0.005763	0.0256051
Var(3)	0.0620826	0.017767	0.02726	0.0969053

Fixed Effects Parameter Estimates

Term	Estimate	Std Error	DFDen	t Ratio	Prob>|t|	95% Lower	95% Upper
Intercept	0.7873896	0.1396337	34.4	5.64	<.0001	0.503756	1.0710231
repeat	-0.054334	0.0254321	27	-2.14	0.0419	-0.106517	-0.002152
clutchsize	-0.026283	0.0156312	27	-1.68	0.1042	-0.058355	0.0057898
(clutchsize-8.31034)*(repeat-2)	0.0195838	0.0152924	27	1.28	0.2112	-0.011794	0.0509612

Fixed Effects Tests

Source	Nparm	DFNum	DFDen	F Ratio	Prob > F
repeat	1	1	27	4.5644383	0.0419
clutchsize	1	1	27	2.8272044	0.1042
clutchsize*repeat	1	1	27	1.6400087	0.2112

Fit Mixed

Actual by Predicted Plot

Fit Statistics

-2 Residual Log Likelihood	-8.909217
-2 Log Likelihood	-40.56728
AICc	-17.67254
BIC	4.0918028

Repeated Effects Covariance Parameter Estimates
Repeated Effect: repeat2

Covariance Parameter	Estimate	Std Error	95% Lower	95% Upper
Var(1)	0.0364455	0.0099112	0.0170199	0.0558712
Cov(2,1)	0.0052203	0.0064325	-0.007387	0.0178278
Var(2)	0.0302396	0.0082017	0.0141646	0.0463145
Cov(3,1)	0.013859	0.0093541	-0.004475	0.0321926
Cov(3,2)	0.0091628	0.0083262	-0.007156	0.0254819
Var(3)	0.0599354	0.0162894	0.0280088	0.091862

Fixed Effects Parameter Estimates

Term	Estimate	Std Error	DFDen	t Ratio	Prob>|t|	95% Lower	95% Upper
Intercept	0.5128906	0.0774093	48.8	6.63	<.0001	0.3573134	0.6684678
repeat	-0.053054	0.02498	27	-2.12	0.043	-0.104309	-0.001799
total_mov	0.0022523	0.0025632	27	0.88	0.3873	-0.003007	0.0075115
(total_mov-22.6552)*(repeat-2)	-0.003275	0.002419	27	-1.35	0.187	-0.008238	0.0016885

Fixed Effects Tests

Source	Nparm	DFNum	DFDen	F Ratio	Prob > F
repeat	1	1	27	4.5107795	0.043
total_mov	1	1	27	0.7721487	0.3873
total_mov*repeat	1	1	27	1.8327944	0.187

Fit Mixed

Actual by Predicted Plot

Fit Statistics

-2 Residual Log Likelihood	-12.44918
-2 Log Likelihood	-41.93782
AICc	-16.41782
BIC	7.1871664

Repeated Effects Covariance Parameter Estimates
Repeated Effect: repeat2

Covariance Parameter	Estimate	Std Error	95% Lower	95% Upper
Var(1)	0.040973	0.0117881	0.0178687	0.0640774
Cov(2,1)	0.0038473	0.0065772	-0.009044	0.0167384
Var(2)	0.0267308	0.0077336	0.0115733	0.0418884
Cov(3,1)	0.0168043	0.0108587	-0.004478	0.0380869
Cov(3,2)	0.0100442	0.0083066	-0.006236	0.0263249
Var(3)	0.0615865	0.0180615	0.0261866	0.0969865

Fixed Effects Parameter Estimates

Term	Estimate	Std Error	DFDen	t Ratio	Prob>|t|	95% Lower	95% Upper
Intercept	0.7867491	0.1421186	33	5.54	<.0001	0.4975936	1.0759047
repeat	-0.054334	0.0254321	27	-2.14	0.0419	-0.106517	-0.002152
age	-0.009224	0.0336467	26	-0.27	0.7861	-0.078386	0.059938
clutchsize	-0.024358	0.0167398	26.1	-1.46	0.1575	-0.058758	0.0100422
(clutchsize-8.31034)*(repeat-2)	0.0195838	0.0152924	27	1.28	0.2112	-0.011793	0.0509612

Fixed Effects Tests

Source	Nparm	DFNum	DFDen	F Ratio	Prob > F
repeat	1	1	27	4.5644394	0.0419
age	1	1	26	0.0751506	0.7861
clutchsize	1	1	26.1	2.1173127	0.1575
clutchsize*repeat	1	1	27	1.6400107	0.2112

Fit Mixed

Actual by Predicted Plot

Fit Statistics

-2 Residual Log Likelihood	-5.053583
-2 Log Likelihood	-41.9058
AICc	-16.3858
BIC	7.2191903

Repeated Effects Covariance Parameter Estimates
Repeated Effect: repeat2

Covariance Parameter	Estimate	Std Error	95% Lower	95% Upper
Var(1)	0.0373629	0.0102536	0.0172663	0.0574596
Cov(2,1)	0.0053024	0.0065091	-0.007455	0.01806
Var(2)	0.0294866	0.0080669	0.0136758	0.0452974
Cov(3,1)	0.0137421	0.009418	-0.004717	0.0322009
Cov(3,2)	0.0082107	0.0081908	-0.007843	0.0242644
Var(3)	0.0587843	0.0160219	0.027382	0.0901865

Fixed Effects Parameter Estimates

Term	Estimate	Std Error	DFDen	t Ratio	Prob>|t|	95% Lower	95% Upper
Intercept	0.5611371	0.0894907	42.6	6.27	<.0001	0.3806126	0.7416615
repeat	-0.053054	0.02498	27	-2.12	0.043	-0.104309	-0.001799
age	-0.034894	0.0322464	26	-1.08	0.2891	-0.101177	0.0313895
total_mov	0.0027349	0.0025671	26.1	1.07	0.2965	-0.002541	0.008011
(total_mov-22.6552)*(repeat-2)	-0.003275	0.002419	27	-1.35	0.187	-0.008238	0.0016885

Fixed Effects Tests

Source	Nparm	DFNum	DFDen	F Ratio	Prob > F
repeat	1	1	27	4.5107739	0.043
age	1	1	26	1.1709459	0.2891
total_mov	1	1	26.1	1.1349472	0.2965
total_mov*repeat	1	1	27	1.8327938	0.187

Fit Mixed

Actual by Predicted Plot

Fit Statistics

-2 Residual Log Likelihood	-12.44918
-2 Log Likelihood	-41.93782
AICc	-16.41782
BIC	7.1871664

Repeated Effects Covariance Parameter Estimates
Repeated Effect: repeat2

Covariance Parameter	Estimate	Std Error	95% Lower	95% Upper
Var(1)	0.040973	0.0117881	0.0178687	0.0640774
Cov(2,1)	0.0038473	0.0065772	-0.009044	0.0167384
Var(2)	0.0267308	0.0077336	0.0115733	0.0418884
Cov(3,1)	0.0168043	0.0108587	-0.004478	0.0380869
Cov(3,2)	0.0100442	0.0083066	-0.006236	0.0263249
Var(3)	0.0615865	0.0180615	0.0261866	0.0969865

Fixed Effects Parameter Estimates

Term	Estimate	Std Error	DFDen	t Ratio	Prob>|t|	95% Lower	95% Upper
Intercept	0.7867491	0.1421186	33	5.54	<.0001	0.4975936	1.0759047
repeat	-0.054334	0.0254321	27	-2.14	0.0419	-0.106517	-0.002152
age	-0.009224	0.0336467	26	-0.27	0.7861	-0.078386	0.059938
clutchsize	-0.024358	0.0167398	26.1	-1.46	0.1575	-0.058758	0.0100422
(clutchsize-8.31034)*(repeat-2)	0.0195838	0.0152924	27	1.28	0.2112	-0.011793	0.0509612

Fixed Effects Tests

Source	Nparm	DFNum	DFDen	F Ratio	Prob > F
repeat	1	1	27	4.5644394	0.0419
age	1	1	26	0.0751506	0.7861
clutchsize	1	1	26.1	2.1173127	0.1575
clutchsize*repeat	1	1	27	1.6400107	0.2112

Fit Mixed

Actual by Predicted Plot

Fit Statistics

-2 Residual Log Likelihood	-11.99084
-2 Log Likelihood	-47.1967
AICc	-21.6767
BIC	1.9282906
Delta AICc	

Repeated Effects Covariance Parameter Estimates
Repeated Effect: repeat2

Covariance Parameter	Estimate	Std Error	95% Lower	95% Upper
Var(1)	0.0406927	0.0118148	0.0175362	0.0638492
Cov(2,1)	-0.000508	0.0065672	-0.013379	0.0123637
Var(2)	0.0259876	0.0074437	0.0113982	0.0405769
Cov(3,1)	0.0178475	0.0106448	-0.003016	0.038711
Cov(3,2)	0.0135771	0.0081952	-0.002485	0.0296394
Var(3)	0.0594633	0.0169111	0.0263182	0.0926083

Fixed Effects Parameter Estimates

Term	Estimate	Std Error	DFDen	t Ratio	Prob>|t|	95% Lower	95% Upper
Intercept	0.7935039	0.1380926	33.5	5.75	<.0001	0.5127265	1.0742814
repeat	-0.057389	0.0246744	25.8	-2.33	0.0281	-0.108123	-0.006656
clutchsize	-0.026283	0.0156311	27	-1.68	0.1042	-0.058356	0.005789
(total_mov-22.6552)*(repeat-2)	-0.005333	0.0023198	26	-2.3	0.0298	-0.010101	-0.000564
(clutchsize-8.31034)*(repeat-2)	0.0285405	0.0148915	25.9	1.92	0.0664	-0.002075	0.0591565

Fixed Effects Tests

Source	Nparm	DFNum	DFDen	F Ratio	Prob > F
repeat	1	1	25.8	5.4096236	0.0281
clutchsize	1	1	27	2.8273707	0.1042
total_mov*repeat	1	1	26	5.2842786	0.0298
clutchsize*repeat	1	1	25.9	3.673222	0.0664

Fit Mixed

Actual by Predicted Plot

Fit Statistics

-2 Residual Log Likelihood	-15.80471
-2 Log Likelihood	-44.19131
AICc	-21.29657
BIC	0.4677713

Repeated Effects Covariance Parameter Estimates
Repeated Effect: repeat2

Covariance Parameter	Estimate	Std Error	95% Lower	95% Upper
Var(1)	0.0370328	0.0101469	0.0171452	0.0569205
Cov(2,1)	0.0021374	0.0059142	-0.009454	0.013729
Var(2)	0.0247099	0.0068278	0.0113278	0.0380921
Cov(3,1)	0.0191029	0.0112415	-0.00293	0.041136
Cov(3,2)	0.0119419	0.0087852	-0.005277	0.0291606
Var(3)	0.0697637	0.0199314	0.0306988	0.1088286

Fixed Effects Parameter Estimates

Term	Estimate	Std Error	DFDen	t Ratio	Prob>|t|	95% Lower	95% Upper
Intercept	0.8685965	0.1357004	32	6.4	<.0001	0.5921704	1.1450226
repeat	-0.053484	0.0250356	26.8	-2.14	0.0419	-0.104872	-0.002096
clutchsize	-0.03611	0.0152825	26.6	-2.36	0.0257	-0.067491	-0.004729
(total_mov-22.6552)*(repeat-2)	-0.004466	0.0023624	26.4	-1.89	0.0697	-0.009318	0.0003866

Fixed Effects Tests

Source	Nparm	DFNum	DFDen	F Ratio	Prob > F
repeat	1	1	26.8	4.5639357	0.0419
clutchsize	1	1	26.6	5.5831091	0.0257
total_mov*repeat	1	1	26.4	3.5732759	0.0697

Fit Mixed

Actual by Predicted Plot

Fit Statistics

-2 Residual Log Likelihood	-6.882719
-2 Log Likelihood	-45.63281
AICc	-20.11281
BIC	3.492184

Repeated Effects Covariance Parameter Estimates
Repeated Effect: repeat2

Covariance Parameter	Estimate	Std Error	95% Lower	95% Upper
Var(1)	0.0382274	0.010657	0.0173401	0.0591146
Cov(2,1)	-0.000965	0.0070375	-0.014759	0.0128279
Var(2)	0.0302398	0.0082018	0.0141646	0.0463149
Cov(3,1)	0.0146421	0.0094718	-0.003922	0.0332064
Cov(3,2)	0.0153486	0.0088993	-0.002094	0.0327909
Var(3)	0.0565878	0.0154189	0.0263672	0.0868083

Fixed Effects Parameter Estimates

Term	Estimate	Std Error	DFDen	t Ratio	Prob>|t|	95% Lower	95% Upper
Intercept	0.5232491	0.0746743	49.3	7.01	<.0001	0.3732071	0.6732911
repeat	-0.058233	0.0247621	25.5	-2.35	0.0267	-0.109181	-0.007286
total_mov	0.0022523	0.0025632	27	0.88	0.3873	-0.003007	0.0075115
(total_mov-22.6552)*(repeat-2)	-0.00457	0.0024067	25.6	-1.9	0.0689	-0.009521	0.0003814
(clutchsize-8.31034)*(repeat-2)	0.0374408	0.0144502	26	2.59	0.0155	0.0077381	0.0671436

Fixed Effects Tests

Source	Nparm	DFNum	DFDen	F Ratio	Prob > F
repeat	1	1	25.5	5.53051	0.0267
total_mov	1	1	27	0.7721413	0.3873
total_mov*repeat	1	1	25.6	3.6053396	0.0689
clutchsize*repeat	1	1	26	6.7134269	0.0155

Fit Mixed

Actual by Predicted Plot

Fit Statistics

-2 Residual Log Likelihood	-13.69461
-2 Log Likelihood	-41.92865
AICc	-19.03392
BIC	2.7304265

Repeated Effects Covariance Parameter Estimates
Repeated Effect: repeat2

Covariance Parameter	Estimate	Std Error	95% Lower	95% Upper
Var(1)	0.0364474	0.0098979	0.0170479	0.055847
Cov(2,1)	0.0009613	0.0068304	-0.012426	0.0143487
Var(2)	0.0296206	0.0079342	0.0140699	0.0451713
Cov(3,1)	0.0147505	0.0097392	-0.004338	0.033839
Cov(3,2)	0.0138711	0.0093333	-0.004422	0.0321639
Var(3)	0.062424	0.0173031	0.0285106	0.0963374

Fixed Effects Parameter Estimates

Term	Estimate	Std Error	DFDen	t Ratio	Prob>|t|	95% Lower	95% Upper
Intercept	0.4821611	0.0749653	39.9	6.43	<.0001	0.3306422	0.63368
repeat	-0.055732	0.0255857	26.4	-2.18	0.0385	-0.108284	-0.003181
total_mov	0.003871	0.0025142	26.2	1.54	0.1356	-0.001295	0.0090372
(clutchsize-8.31034)*(repeat-2)	0.0306931	0.0150036	26.2	2.05	0.0509	-0.000134	0.0615198

Fixed Effects Tests

Source	Nparm	DFNum	DFDen	F Ratio	Prob > F
repeat	1	1	26.4	4.7448133	0.0385
total_mov	1	1	26.2	2.3705148	0.1356
clutchsize*repeat	1	1	26.2	4.1849328	0.0509

Fit Mixed

Actual by Predicted Plot

Fit Statistics

-2 Residual Log Likelihood	-16.26495
-2 Log Likelihood	-44.85572
AICc	-21.96098
BIC	-0.196637
Delta AICc	

Repeated Effects Covariance Parameter Estimates
Repeated Effect: repeat2

Covariance Parameter	Estimate	Std Error	95% Lower	95% Upper
Var(1)	0.0372693	0.0102564	0.0171672	0.0573715
Cov(2,1)	-0.001346	0.0071106	-0.015283	0.0125904
Var(2)	0.0312786	0.0083118	0.0149877	0.0475696
Cov(3,1)	0.0132859	0.009054	-0.00446	0.0310314
Cov(3,2)	0.0154593	0.008893	-0.001971	0.0328893
Var(3)	0.0550222	0.014726	0.0261598	0.0838847

Fixed Effects Parameter Estimates

Term	Estimate	Std Error	DFDen	t Ratio	Prob>|t|	95% Lower	95% Upper
Intercept	0.5740127	0.0469718	25.9	12.22	<.0001	0.4774374	0.670588
repeat	-0.058509	0.0247587	25.6	-2.36	0.026	-0.109443	-0.007575
(total_mov-22.6552)*(repeat-2)	-0.005333	0.002328	26	-2.29	0.0303	-0.010118	-0.000547

(clutchsize-8.31034)*(repeat-2)	0.0374409	0.0144556	26	2.59	0.0155	0.007727	0.0671548

Fixed Effects Tests

Source	Nparm	DFNum	DFDen	F Ratio	Prob > F
repeat	1	1	25.6	5.5845661	0.026
total_mov*repeat	1	1	26	5.2469473	0.0303
clutchsize*repeat	1	1	26	6.7084249	0.0155

Fit Mixed

Actual by Predicted Plot

Fit Statistics

-2 Residual Log Likelihood	-21.76977
-2 Log Likelihood	-39.60348
AICc	-19.26582
BIC	0.5896916

Repeated Effects Covariance Parameter Estimates
Repeated Effect: repeat2

Covariance Parameter	Estimate	Std Error	95% Lower	95% Upper
Var(1)	0.0371545	0.0100256	0.0175047	0.0568043
Cov(2,1)	0.0030959	0.0069296	-0.010486	0.0166777
Var(2)	0.0312787	0.0083119	0.0149877	0.0475696
Cov(3,1)	0.0125024	0.0089989	-0.005135	0.03014
Cov(3,2)	0.0110173	0.0085901	-0.005819	0.0278537
Var(3)	0.056704	0.0151528	0.027005	0.086403

Fixed Effects Parameter Estimates

Term	Estimate	Std Error	DFDen	t Ratio	Prob>|t|	95% Lower	95% Upper
Intercept	0.5672285	0.0513083	26.8	11.06	<.0001	0.4619214	0.6725356
repeat	-0.055117	0.0254726	26.9	-2.16	0.0395	-0.107395	-0.002839
(clutchsize-8.31034)*(repeat-2)	0.0258023	0.0153687	27	1.68	0.1047	-0.005732	0.0573363

Fixed Effects Tests

Source	Nparm	DFNum	DFDen	F Ratio	Prob > F
repeat	1	1	26.9	4.681924	0.0395
clutchsize*repeat	1	1	27	2.8186383	0.1047

Fit Mixed

Actual by Predicted Plot

Fit Statistics

-2 Residual Log Likelihood	-18.29144
-2 Log Likelihood	-39.79017
AICc	-19.45251
BIC	0.403003

Repeated Effects Covariance Parameter Estimates
Repeated Effect: repeat2

Covariance Parameter	Estimate	Std Error	95% Lower	95% Upper
Var(1)	0.0355799	0.0095182	0.0169245	0.0542353
Cov(2,1)	0.005088	0.0064639	-0.007581	0.0177571
Var(2)	0.0312787	0.0083119	0.0149877	0.0475696
Cov(3,1)	0.0125951	0.0089188	-0.004885	0.0300756
Cov(3,2)	0.0090252	0.0082884	-0.00722	0.0252701
Var(3)	0.0580932	0.0154947	0.0277241	0.0884623

Fixed Effects Parameter Estimates

Term	Estimate	Std Error	DFDen	t Ratio	Prob>|t|	95% Lower	95% Upper
Intercept	0.5634377	0.0511635	27	11.01	<.0001	0.4584667	0.6684087
repeat	-0.053221	0.0249746	27	-2.13	0.0423	-0.104463	-0.00198
(total_mov-22.6552)*(repeat-2)	-0.003738	0.0024404	27	-1.53	0.1372	-0.008745	0.0012691

Fixed Effects Tests

Source	Nparm	DFNum	DFDen	F Ratio	Prob > F
repeat	1	1	27	4.5412709	0.0423
total_mov*repeat	1	1	27	2.3463387	0.1372

Minimum distance Fit Group

Fit Mixed

Actual by Predicted Plot

Fit Statistics

-2 Residual Log Likelihood	461.21141
-2 Log Likelihood	453.03003
AICc	484.1568
BIC	510.7845

Repeated Effects Covariance Parameter Estimates
Repeated Effect: repeat2

Covariance Parameter	Estimate	Std Error	95% Lower	95% Upper
Var(1)	17.887468	4.959108	8.167795	27.607141
Cov(2,1)	9.6579652	3.7967616	2.2164491	17.099481
Var(2)	15.127844	4.2076941	6.8809155	23.374773
Cov(3,1)	15.061593	5.0869709	5.091313	25.031873
Cov(3,2)	8.2315163	4.3178595	-0.231333	16.694365
Var(3)	25.39534	7.0294114	11.617946	39.172733

Fixed Effects Parameter Estimates

Term	Estimate	Std Error	DFDen	t Ratio	Prob>|t|	95% Lower	95% Upper
Intercept	6.4384873	3.7424235	26.3	1.72	0.0971	-1.249544	14.126518
repeat	1.1237432	0.3356719	25.2	3.35	0.0026	0.4326311	1.8148553
age	-0.38328	0.9031289	25.1	-0.42	0.6749	-2.243109	1.4765483
clutchsize	-0.104845	0.4359819	25	-0.24	0.8119	-1.002841	0.7931509
total_mov	-0.039639	0.0672225	24.8	-0.59	0.5607	-0.178132	0.0988548
(total_mov-22.6235)*(repeat-1.97647)	-0.042925	0.0319309	24.7	-1.34	0.1911	-0.108727	0.0228779
(clutchsize-8.32941)*(repeat-1.97647)	0.0842138	0.1988088	24.8	0.42	0.6755	-0.325435	0.4938631

Fixed Effects Tests

Source	Nparm	DFNum	DFDen	F Ratio	Prob > F
repeat	1	1	25.2	11.207379	0.0026
age	1	1	25.1	0.1801081	0.6749
clutchsize	1	1	25	0.0578306	0.8119
total_mov	1	1	24.8	0.3477007	0.5607
total_mov*repeat	1	1	24.7	1.8071422	0.1911
clutchsize*repeat	1	1	24.8	0.1794301	0.6755

Fit Mixed

Actual by Predicted Plot

Fit Statistics

-2 Residual Log Likelihood	462.90181
-2 Log Likelihood	453.12992
AICc	481.46326
BIC	506.44174

Repeated Effects Covariance Parameter Estimates
Repeated Effect: repeat2

Covariance Parameter	Estimate	Std Error	95% Lower	95% Upper
Var(1)	17.377905	4.7610657	8.0463874	26.709422
Cov(2,1)	9.2379597	3.618363	2.1460986	16.329821
Var(2)	14.797463	4.054247	6.8512854	22.743642
Cov(3,1)	14.629189	4.9089477	5.0078284	24.25055
Cov(3,2)	7.8912186	4.1578947	-0.258105	16.040542
Var(3)	25.036255	6.879186	11.553298	38.519211

Fixed Effects Parameter Estimates

Term	Estimate	Std Error	DFDen	t Ratio	Prob>|t|	95% Lower	95% Upper
Intercept	6.3806492	3.6837908	27.4	1.73	0.0945	-1.172236	13.933534
repeat	1.1246416	0.335653	25.2	3.35	0.0025	0.4335667	1.8157166
clutchsize	-0.165015	0.4069951	25.9	-0.41	0.6885	-1.001796	0.6717671
total_mov	-0.043721	0.0655385	25.9	-0.67	0.5106	-0.178471	0.0910289
(total_mov-22.6235)*(repeat-1.97647)	-0.042822	0.03193	24.7	-1.34	0.1921	-0.108623	0.0229786
(clutchsize-8.32941)*(repeat-1.97647)	0.0835269	0.1988023	24.8	0.42	0.678	-0.326109	0.4931628

Fixed Effects Tests

Source	Nparm	DFNum	DFDen	F Ratio	Prob > F
repeat	1	1	25.2	11.226575	0.0025
clutchsize	1	1	25.9	0.1643868	0.6885
total_mov	1	1	25.9	0.4450316	0.5106
total_mov*repeat	1	1	24.7	1.7986112	0.1921
clutchsize*repeat	1	1	24.8	0.1765265	0.678

Fit Mixed

Actual by Predicted Plot

Fit Statistics

-2 Residual Log Likelihood	461.60762
-2 Log Likelihood	453.24319
AICc	478.85963
BIC	502.11235

Repeated Effects Covariance Parameter Estimates
Repeated Effect: repeat2

Covariance Parameter	Estimate	Std Error	95% Lower	95% Upper
Var(1)	17.363586	4.750604	8.0525735	26.674599
Cov(2,1)	9.2206976	3.6127906	2.1397582	16.301637
Var(2)	14.804038	4.0574566	6.8515695	22.756507
Cov(3,1)	14.748813	4.9014197	5.1422065	24.355419
Cov(3,2)	7.9522967	4.1263467	-0.135194	16.039788
Var(3)	24.821046	6.7794765	11.533516	38.108576

Fixed Effects Parameter Estimates

Term	Estimate	Std Error	DFDen	t Ratio	Prob>|t|	95% Lower	95% Upper
Intercept	6.4554346	3.7830892	27.3	1.71	0.0993	-1.302307	14.213176
repeat	1.1249652	0.3301472	26.1	3.41	0.0021	0.4465128	1.8034175
clutchsize	-0.17445	0.4211923	25.9	-0.41	0.6821	-1.040322	0.6914217
total_mov	-0.0435	0.0655882	25.9	-0.66	0.513	-0.178348	0.0913486
(total_mov-22.6235)*(repeat-1.97647)	-0.041508	0.0312397	25.7	-1.33	0.1956	-0.105763	0.0227469

Fixed Effects Tests

Source	Nparm	DFNum	DFDen	F Ratio	Prob > F
repeat	1	1	26.1	11.610823	0.0021
clutchsize	1	1	25.9	0.1715466	0.6821
total_mov	1	1	25.9	0.4398697	0.513
total_mov*repeat	1	1	25.7	1.7654484	0.1956

Fit Mixed

Actual by Predicted Plot

Fit Statistics

-2 Residual Log Likelihood	459.69671
-2 Log Likelihood	455.12378
AICc	480.74022
BIC	503.99294

Repeated Effects Covariance Parameter Estimates
Repeated Effect: repeat2

Covariance Parameter	Estimate	Std Error	95% Lower	95% Upper
Var(1)	17.424559	4.7826422	8.0507525	26.798365
Cov(2,1)	9.2391524	3.6255079	2.1332874	16.345017
Var(2)	14.797932	4.0545519	6.851156	22.744707
Cov(3,1)	14.580995	4.9312124	4.9159963	24.245994
Cov(3,2)	7.9613203	4.2022896	-0.275016	16.197657
Var(3)	25.347918	6.9997381	11.628683	39.067153

Fixed Effects Parameter Estimates

Term	Estimate	Std Error	DFDen	t Ratio	Prob>|t|	95% Lower	95% Upper
Intercept	6.2921854	3.7034441	27.5	1.7	0.1006	-1.300302	13.884673
repeat	1.129267	0.3415574	26.2	3.31	0.0027	0.4274226	1.8311114
clutchsize	-0.168868	0.4075552	25.9	-0.41	0.682	-1.006821	0.6690853
total_mov	-0.038655	0.0680336	25.9	-0.57	0.5748	-0.178519	0.1012092
(clutchsize-8.32941)*(repeat-1.97647)	0.0568747	0.2012532	25.7	0.28	0.7797	-0.35701	0.4707597

Fixed Effects Tests

Source	Nparm	DFNum	DFDen	F Ratio	Prob > F
repeat	1	1	26.2	10.93115	0.0027
clutchsize	1	1	25.9	0.1716802	0.682
total_mov	1	1	25.9	0.3228244	0.5748
clutchsize*repeat	1	1	25.7	0.0798643	0.7797

Fit Mixed

Actual by Predicted Plot

Fit Statistics

-2 Residual Log Likelihood	456.35164
-2 Log Likelihood	455.41615
AICc	478.38912
BIC	499.84266

Repeated Effects Covariance Parameter Estimates
Repeated Effect: repeat2

Covariance Parameter	Estimate	Std Error	95% Lower	95% Upper
Var(1)	16.843359	4.5521276	7.9213533	25.765366
Cov(2,1)	8.8203377	3.4502301	2.0580109	15.582664
Var(2)	14.541426	3.9314196	6.8359857	22.246867
Cov(3,1)	14.397996	4.8191078	4.9527187	23.843274
Cov(3,2)	7.9621387	4.1021458	-0.077919	16.002197
Var(3)	25.568059	6.9716266	11.903922	39.232196

Fixed Effects Parameter Estimates

Term	Estimate	Std Error	DFDen	t Ratio	Prob>|t|	95% Lower	95% Upper
Intercept	5.5871314	3.4571574	28.8	1.62	0.117	-1.485769	12.660031
repeat	1.1325109	0.341796	26.1	3.31	0.0027	0.4301002	1.8349215
clutchsize	-0.189862	0.4019779	26.9	-0.47	0.6405	-1.014838	0.635114
(clutchsize-8.32941)*(repeat-1.97647)	0.0555679	0.2013815	25.7	0.28	0.7848	-0.358624	0.4697602

Fixed Effects Tests

Source	Nparm	DFNum	DFDen	F Ratio	Prob > F
repeat	1	1	26.1	10.978699	0.0027
clutchsize	1	1	26.9	0.2230853	0.6405
clutchsize*repeat	1	1	25.7	0.0761392	0.7848

Fit Mixed

Actual by Predicted Plot

Fit Statistics

-2 Residual Log Likelihood	461.75647
-2 Log Likelihood	453.36084
AICc	476.33381
BIC	497.78735

Repeated Effects Covariance Parameter Estimates
Repeated Effect: repeat2

Covariance Parameter	Estimate	Std Error	95% Lower	95% Upper
Var(1)	17.20127	4.6367711	8.1133656	26.289174
Cov(2,1)	8.8915727	3.4627943	2.1046205	15.678525
Var(2)	14.309171	3.8601956	6.7433265	21.875015
Cov(3,1)	14.544364	4.7895636	5.1569919	23.931736
Cov(3,2)	7.5716481	3.9792502	-0.227539	15.370835
Var(3)	24.574033	6.6775329	11.486309	37.661757

Fixed Effects Parameter Estimates

Term	Estimate	Std Error	DFDen	t Ratio	Prob>|t|	95% Lower	95% Upper
Intercept	5.0662778	1.7148782	33.9	2.95	0.0057	1.5809855	8.5515701
repeat	1.123442	0.3300773	26.2	3.4	0.0022	0.4451583	1.8017256
total_mov	-0.045899	0.0642617	26.8	-0.71	0.4812	-0.177788	0.0859901
(total_mov-22.6235)*(repeat-1.97647)	-0.041547	0.0312332	25.7	-1.33	0.1951	-0.105786	0.0226925

Fixed Effects Tests

Source	Nparm	DFNum	DFDen	F Ratio	Prob > F
repeat	1	1	26.2	11.584308	0.0022
total_mov	1	1	26.8	0.5101509	0.4812
total_mov*repeat	1	1	25.7	1.7694805	0.1951

Fit Mixed

Actual by Predicted Plot

Fit Statistics

-2 Residual Log Likelihood	454.6471
-2 Log Likelihood	455.21816
AICc	480.8346
BIC	504.08732

Repeated Effects Covariance Parameter Estimates
Repeated Effect: repeat2

Covariance Parameter	Estimate	Std Error	95% Lower	95% Upper
Var(1)	17.33436	4.7409991	8.0421722	26.626547
Cov(2,1)	9.1952324	3.6090293	2.121665	16.2688
Var(2)	14.800024	4.0589858	6.844558	22.75549
Cov(3,1)	14.782843	4.980196	5.0218385	24.543848
Cov(3,2)	8.2268439	4.237103	-0.077725	16.531413
Var(3)	25.849221	7.0979471	11.937501	39.760942

Fixed Effects Parameter Estimates

Term	Estimate	Std Error	DFDen	t Ratio	Prob>|t|	95% Lower	95% Upper
Intercept	5.7324828	3.5166	27.7	1.63	0.1144	-1.474997	12.939963
repeat	1.1312823	0.3417828	26.1	3.31	0.0027	0.4289086	1.833656
age	-0.425408	0.8810028	26	-0.48	0.6332	-2.236176	1.3853589
clutchsize	-0.120432	0.4308268	26	-0.28	0.782	-1.006037	0.7651732
(clutchsize-8.32941)*(repeat-1.97647)	0.0562919	0.2013709	25.7	0.28	0.7821	-0.357874	0.4704575

Fixed Effects Tests

Source	Nparm	DFNum	DFDen	F Ratio	Prob > F
repeat	1	1	26.1	10.955733	0.0027
age	1	1	26	0.2331619	0.6332
clutchsize	1	1	26	0.0781406	0.782
clutchsize*repeat	1	1	25.7	0.0781445	0.7821

Fit Mixed

Actual by Predicted Plot

Fit Statistics

-2 Residual Log Likelihood	460.07508
-2 Log Likelihood	453.09084
AICc	478.70728
BIC	501.96001

Repeated Effects Covariance Parameter Estimates
Repeated Effect: repeat2

Covariance Parameter	Estimate	Std Error	95% Lower	95% Upper
Var(1)	17.562601	4.7808352	8.1923365	26.932866
Cov(2,1)	9.2160985	3.6076535	2.1452275	16.28697
Var(2)	14.597675	3.9925278	6.7724643	22.422886
Cov(3,1)	14.842729	4.9229339	5.1939559	24.491502
Cov(3,2)	7.8335548	4.1104929	-0.222863	15.889973
Var(3)	24.812362	6.7911763	11.501901	38.122823

Fixed Effects Parameter Estimates

Term	Estimate	Std Error	DFDen	t Ratio	Prob>|t|	95% Lower	95% Upper
Intercept	5.7002138	2.0988675	30.7	2.72	0.0108	1.4177919	9.9826356
repeat	1.1226964	0.3301115	26.2	3.4	0.0022	0.4443397	1.8010531
age	-0.452101	0.8430435	26	-0.54	0.5963	-2.185004	1.2808024
total_mov	-0.040106	0.0659472	25.8	-0.61	0.5484	-0.175708	0.0954957
(total_mov-22.6235)*(repeat-1.97647)	-0.041645	0.0312358	25.7	-1.33	0.1942	-0.10589	0.0226003

Fixed Effects Tests

Source	Nparm	DFNum	DFDen	F Ratio	Prob > F
repeat	1	1	26.2	11.566541	0.0022
age	1	1	26	0.2875879	0.5963
total_mov	1	1	25.8	0.3698491	0.5484
total_mov*repeat	1	1	25.7	1.7775498	0.1942

Fit Mixed

Actual by Predicted Plot

Fit Statistics

-2 Residual Log Likelihood	454.6471
-2 Log Likelihood	455.21816
AICc	480.8346
BIC	504.08732

Repeated Effects Covariance Parameter Estimates
Repeated Effect: repeat2

Covariance Parameter	Estimate	Std Error	95% Lower	95% Upper
Var(1)	17.33436	4.7409991	8.0421722	26.626547
Cov(2,1)	9.1952324	3.6090293	2.121665	16.2688
Var(2)	14.800024	4.0589858	6.844558	22.75549

Cov(3,1)	14.782843	4.980196	5.0218385	24.543848
Cov(3,2)	8.2268439	4.237103	-0.077725	16.531413
Var(3)	25.849221	7.0979471	11.937501	39.760942

Fixed Effects Parameter Estimates

Term	Estimate	Std Error	DFDen	t Ratio	Prob>|t|	95% Lower	95% Upper
Intercept	5.7324828	3.5166	27.7	1.63	0.1144	-1.474997	12.939963
repeat	1.1312823	0.3417828	26.1	3.31	0.0027	0.4289086	1.833656
age	-0.425408	0.8810028	26	-0.48	0.6332	-2.236176	1.3853589
clutchsize	-0.120432	0.4308268	26	-0.28	0.782	-1.006037	0.7651732
(clutchsize-8.32941)*(repeat-1.97647)	0.0562919	0.2013709	25.7	0.28	0.7821	-0.357874	0.4704575

Fixed Effects Tests

Source	Nparm	DFNum	DFDen	F Ratio	Prob > F
repeat	1	1	26.1	10.955733	0.0027
age	1	1	26	0.2331619	0.6332
clutchsize	1	1	26	0.0781406	0.782
clutchsize*repeat	1	1	25.7	0.0781445	0.7821

Fit Mixed

Actual by Predicted Plot

Fit Statistics

-2 Residual Log Likelihood	459.69194
-2 Log Likelihood	453.56186
AICc	479.1783
BIC	502.43102

Repeated Effects Covariance Parameter Estimates
Repeated Effect: repeat2

Covariance Parameter	Estimate	Std Error	95% Lower	95% Upper
Var(1)	17.18137	4.65713	8.0535631	26.309177
Cov(2,1)	9.0070039	3.4959245	2.1551178	15.85889
Var(2)	14.530622	3.9280834	6.8317199	22.229524
Cov(3,1)	14.438147	4.8088303	5.0130127	23.863281
Cov(3,2)	7.6623186	4.0338982	-0.243977	15.568614
Var(3)	24.849139	6.7810305	11.558563	38.139715

Fixed Effects Parameter Estimates

Term	Estimate	Std Error	DFDen	t Ratio	Prob>|t|	95% Lower	95% Upper
Intercept	5.5856728	3.4552717	28.7	1.62	0.1169	-1.483987	12.655333
repeat	1.1248113	0.3355911	25.2	3.35	0.0025	0.4338832	1.8157393
clutchsize	-0.188449	0.4014379	26.9	-0.47	0.6425	-1.012306	0.6354077
(total_mov-22.6235)*(repeat-1.97647)	-0.041702	0.0330461	24.8	-1.26	0.2187	-0.109783	0.026379
(clutchsize-8.32941)*(repeat-1.97647)	0.0826331	0.1988239	24.8	0.42	0.6813	-0.327033	0.4922989

Fixed Effects Tests

Source	Nparm	DFNum	DFDen	F Ratio	Prob > F
repeat	1	1	25.2	11.234101	0.0025
clutchsize	1	1	26.9	0.220369	0.6425
total_mov*repeat	1	1	24.8	1.59248	0.2187
clutchsize*repeat	1	1	24.8	0.1727315	0.6813

Fit Mixed

Actual by Predicted Plot

Fit Statistics

-2 Residual Log Likelihood	458.39337
-2 Log Likelihood	453.66962
AICc	476.64259
BIC	498.09613

Repeated Effects Covariance Parameter Estimates
Repeated Effect: repeat2

Covariance Parameter	Estimate	Std Error	95% Lower	95% Upper
Var(1)	17.153395	4.6438535	8.0516094	26.255181
Cov(2,1)	8.9834813	3.4884394	2.1462656	15.820697
Var(2)	14.537045	3.9311451	6.8321425	22.241948
Cov(3,1)	14.554797	4.8003848	5.146216	23.963378
Cov(3,2)	7.7270333	4.0029107	-0.118527	15.572594
Var(3)	24.643088	6.6848645	11.540994	37.745182

Fixed Effects Parameter Estimates

Term	Estimate	Std Error	DFDen	t Ratio	Prob>|t|	95% Lower	95% Upper
Intercept	5.6638776	3.5626274	28.6	1.59	0.1229	-1.627479	12.955234
repeat	1.1252067	0.3300946	26.2	3.41	0.0021	0.4468788	1.8035345
clutchsize	-0.197713	0.4150267	26.9	-0.48	0.6376	-1.049369	0.6539424
(total_mov-22.6235)*(repeat-1.97647)	-0.04037	0.0322855	25.8	-1.25	0.2224	-0.106759	0.0260188

Fixed Effects Tests

Source	Nparm	DFNum	DFDen	F Ratio	Prob > F
repeat	1	1	26.2	11.619507	0.0021
clutchsize	1	1	26.9	0.226944	0.6376
total_mov*repeat	1	1	25.8	1.5635216	0.2224

Fit Mixed

Actual by Predicted Plot

Fit Statistics

-2 Residual Log Likelihood	463.03288
-2 Log Likelihood	453.22656
AICc	478.843
BIC	502.09572

Repeated Effects Covariance Parameter Estimates
Repeated Effect: repeat2

Covariance Parameter	Estimate	Std Error	95% Lower	95% Upper
Var(1)	17.139803	4.6342783	8.0567844	26.222822
Cov(2,1)	8.8778815	3.4570307	2.1022259	15.653537
Var(2)	14.309777	3.8603501	6.7436294	21.875924
Cov(3,1)	14.415086	4.7909426	5.0250109	23.805161
Cov(3,2)	7.5421537	4.0003658	-0.298419	15.382727
Var(3)	24.851156	6.7666197	11.588825	38.113487

Fixed Effects Parameter Estimates

Term	Estimate	Std Error	DFDen	t Ratio	Prob>|t|	95% Lower	95% Upper
Intercept	5.06594	1.7170331	34.1	2.95	0.0057	1.5769136	8.5549665
repeat	1.1236065	0.3355716	25.2	3.35	0.0026	0.432731	1.8144821
total_mov	-0.04599	0.0642285	26.8	-0.72	0.4802	-0.177812	0.0858329
(total_mov-22.6235)*(repeat-1.97647)	-0.042921	0.031932	24.7	-1.34	0.1911	-0.108723	0.0228804
(clutchsize-8.32941)*(repeat-1.97647)	0.0879639	0.2056254	24.9	0.43	0.6725	-0.335607	0.5115344

Fixed Effects Tests

Source	Nparm	DFNum	DFDen	F Ratio	Prob > F
repeat	1	1	25.2	11.211359	0.0026
total_mov	1	1	26.8	0.5126982	0.4802
total_mov*repeat	1	1	24.7	1.8067176	0.1911
clutchsize*repeat	1	1	24.9	0.1830019	0.6725

Fit Mixed

Actual by Predicted Plot

Fit Statistics

-2 Residual Log Likelihood	459.83743
-2 Log Likelihood	455.22905
AICc	478.20202
BIC	499.65556

Repeated Effects Covariance Parameter Estimates
Repeated Effect: repeat2

Covariance Parameter	Estimate	Std Error	95% Lower	95% Upper
Var(1)	17.215943	4.6606238	8.0812882	26.350598
Cov(2,1)	8.8922676	3.466985	2.097102	15.687433
Var(2)	14.306336	3.8587598	6.7433057	21.869366
Cov(3,1)	14.371307	4.8154724	4.9331545	23.809459
Cov(3,2)	7.5986282	4.0416581	-0.322876	15.520133
Var(3)	25.142702	6.8850521	11.648248	38.637157

Fixed Effects Parameter Estimates

Term	Estimate	Std Error	DFDen	t Ratio	Prob>|t|	95% Lower	95% Upper
Intercept	4.9558659	1.7662893	33.4	2.81	0.0083	1.3639628	8.547769
repeat	1.1280232	0.3414614	26.2	3.3	0.0028	0.4264107	1.8296357
total_mov	-0.041367	0.0665984	26.9	-0.62	0.5397	-0.178038	0.0953045
(clutchsize-8.32941)*(repeat-1.97647)	0.0617646	0.2083296	25.9	0.3	0.7692	-0.366555	0.4900845

Fixed Effects Tests

Source	Nparm	DFNum	DFDen	F Ratio	Prob > F
repeat	1	1	26.2	10.913221	0.0028
total_mov	1	1	26.9	0.3858135	0.5397
clutchsize*repeat	1	1	25.9	0.0878976	0.7692

Fit Mixed

Actual by Predicted Plot

Fit Statistics

-2 Residual Log Likelihood	459.85664
-2 Log Likelihood	453.75442
AICc	476.72739
BIC	498.18093

Repeated Effects Covariance Parameter Estimates
Repeated Effect: repeat2

Covariance Parameter	Estimate	Std Error	95% Lower	95% Upper
Var(1)	17.008101	4.5553403	8.079798	25.936404
Cov(2,1)	8.6940981	3.3528636	2.1226063	15.26559
Var(2)	14.07156	3.7473514	6.7268863	21.416234
Cov(3,1)	14.299457	4.7155308	5.0571869	23.541728
Cov(3,2)	7.3705071	3.8964308	-0.266357	15.007371
Var(3)	24.751186	6.6994608	11.620484	37.881888

Fixed Effects Parameter Estimates

Term	Estimate	Std Error	DFDen	t Ratio	Prob>|t|	95% Lower	95% Upper
Intercept	4.0253825	0.9063821	27.3	4.44	0.0001	2.1665667	5.8841984
repeat	1.1238314	0.3355215	25.2	3.35	0.0026	0.4330745	1.8145882
(total_mov-22.6235)*(repeat-1.97647)	-0.041764	0.0330112	24.9	-1.27	0.2176	-0.109772	0.026244
(clutchsize-8.32941)*(repeat-1.97647)	0.0876358	0.2054876	24.9	0.43	0.6734	-0.335644	0.5109152

Fixed Effects Tests

Source	Nparm	DFNum	DFDen	F Ratio	Prob > F
repeat	1	1	25.2	11.219194	0.0026
total_mov*repeat	1	1	24.9	1.6006049	0.2176
clutchsize*repeat	1	1	24.9	0.1818828	0.6734

Fit Mixed

Actual by Predicted Plot

Fit Statistics

-2 Residual Log Likelihood	456.52177
-2 Log Likelihood	455.61192
AICc	476.01192
BIC	495.59579

Repeated Effects Covariance Parameter Estimates
Repeated Effect: repeat2

Covariance Parameter	Estimate	Std Error	95% Lower	95% Upper
Var(1)	16.683169	4.4563009	7.9489796	25.417358
Cov(2,1)	8.5158686	3.3100481	2.0282936	15.003444
Var(2)	14.079892	3.749488	6.7310304	21.428753
Cov(3,1)	14.264001	4.7269282	4.9993922	23.52861
Cov(3,2)	7.6653855	3.9615567	-0.099123	15.429894
Var(3)	25.468989	6.8869897	11.970738	38.967241

Fixed Effects Parameter Estimates

Term	Estimate	Std Error	DFDen	t Ratio	Prob>|t|	95% Lower	95% Upper
Intercept	4.014798	0.8967646	27.8	4.48	0.0001	2.1773739	5.8522221
repeat	1.1316871	0.3416973	26.2	3.31	0.0027	0.4295195	1.8338548

(clutchsize-8.32941)*(repeat-1.97647)	0.0643744	0.2074717	25.9	0.31	0.7588	-0.362157	0.4909059

Fixed Effects Tests

Source	Nparm	DFNum	DFDen	F Ratio	Prob > F
repeat	1	1	26.2	10.969063	0.0027
clutchsize*repeat	1	1	25.9	0.0962738	0.7588

Fit Mixed

Actual by Predicted Plot

Fit Statistics

-2 Residual Log Likelihood	458.57852
-2 Log Likelihood	453.88604
AICc	474.28604
BIC	493.8699

Repeated Effects Covariance Parameter Estimates
Repeated Effect: repeat2

Covariance Parameter	Estimate	Std Error	95% Lower	95% Upper
Var(1)	17.066624	4.5569375	8.1351909	25.998058
Cov(2,1)	8.7062217	3.358071	2.1245235	15.28792
Var(2)	14.070639	3.7470332	6.7265891	21.414689
Cov(3,1)	14.425755	4.7134408	5.1875803	23.663929
Cov(3,2)	7.3980876	3.8752033	-0.197171	14.993346
Var(3)	24.4714	6.6089949	11.518008	37.424792

Fixed Effects Parameter Estimates

Term	Estimate	Std Error	DFDen	t Ratio	Prob>|t|	95% Lower	95% Upper
Intercept	4.0277376	0.9010969	27.6	4.47	0.0001	2.1807292	5.8747459
repeat	1.1236536	0.3300283	26.2	3.4	0.0021	0.4454848	1.8018223
(total_mov-22.6235)*(repeat-1.97647)	-0.040507	0.0323073	25.8	-1.25	0.2212	-0.10694	0.0259268

Fixed Effects Tests

Source	Nparm	DFNum	DFDen	F Ratio	Prob > F
repeat	1	1	26.2	11.59211	0.0021
total_mov*repeat	1	1	25.8	1.5720037	0.2212

Latency Fit Group

Fit Mixed

Actual by Predicted Plot

Fit Statistics

-2 Residual Log Likelihood	809.80546
-2 Log Likelihood	829.14497
AICc	860.13127
BIC	887.20178

Repeated Effects Covariance Parameter Estimates
Repeated Effect: repeat2

Covariance Parameter	Estimate	Std Error	95% Lower	95% Upper
Var(1)	1990.1567	575.19123	862.80264	3117.5108
Cov(2,1)	88.296678	146.63475	-199.1021	375.6955
Var(2)	246.38616	70.681246	107.85346	384.91886
Cov(3,1)	290.96409	336.4939	-368.5518	950.48002
Cov(3,2)	-12.16784	119.79003	-246.952	222.61631
Var(3)	1428.0922	409.15098	626.171	2230.0134

Fixed Effects Parameter Estimates

Term	Estimate	Std Error	DFDen	t Ratio	Prob>|t|	95% Lower	95% Upper
Intercept	38.674039	18.57895	40.5	2.08	0.0437	1.1386539	76.209424
repeat	-2.519517	5.1378029	26	-0.49	0.628	-13.08042	8.0413856
age	5.0153619	3.6629956	25	1.37	0.1831	-2.528627	12.559351
clutchsize	-3.994932	1.7863469	25.1	-2.24	0.0345	-7.673474	-0.316389
total_mov	0.2168558	0.275842	25	0.79	0.4392	-0.351299	0.7850102
(total_mov-22.6552)*(repeat-2)	-0.022241	0.4994908	26	-0.04	0.9648	-1.048958	1.0044771
(clutchsize-8.31034)*(repeat-2)	-2.788806	3.1015595	26	-0.9	0.3768	-9.164152	3.5865389

Fixed Effects Tests

Source	Nparm	DFNum	DFDen	F Ratio	Prob > F
repeat	1	1	26	0.2404804	0.628
age	1	1	25	1.8747	0.1831
clutchsize	1	1	25.1	5.0013484	0.0345
total_mov	1	1	25	0.6180469	0.4392
total_mov*repeat	1	1	26	0.0019826	0.9648
clutchsize*repeat	1	1	26	0.8084935	0.3768

Fit Mixed

Actual by Predicted Plot

Fit Statistics

-2 Residual Log Likelihood	816.02043
-2 Log Likelihood	831.19767
AICc	859.41389
BIC	884.78857

Repeated Effects Covariance Parameter Estimates
Repeated Effect: repeat2

Covariance Parameter	Estimate	Std Error	95% Lower	95% Upper
Var(1)	1884.6417	538.94301	828.33282	2940.9506
Cov(2,1)	41.680505	139.26484	-231.2736	314.63458
Var(2)	258.70644	73.44409	114.75867	402.65421
Cov(3,1)	253.45425	328.72522	-390.8353	897.74384
Cov(3,2)	9.2543265	122.55367	-230.9464	249.4551
Var(3)	1458.6007	417.7715	639.78361	2277.4178

Fixed Effects Parameter Estimates

Term	Estimate	Std Error	DFDen	t Ratio	Prob>|t|	95% Lower	95% Upper
Intercept	40.262843	18.509727	43.3	2.18	0.0351	2.9422159	77.583471
repeat	-2.519368	5.1378095	26	-0.49	0.628	-13.08029	8.0415498
clutchsize	-3.31582	1.7051807	26	-1.94	0.0627	-6.820844	0.1892042
total_mov	0.2845475	0.2746109	26	1.04	0.3097	-0.279919	0.8490142
(total_mov-22.6552)*(repeat-2)	-0.022234	0.4994914	26	-0.04	0.9648	-1.048953	1.0044857
(clutchsize-8.31034)*(repeat-2)	-2.788853	3.1015635	26	-0.9	0.3768	-9.164208	3.5865018

Fixed Effects Tests

Source	Nparm	DFNum	DFDen	F Ratio	Prob > F
repeat	1	1	26	0.2404514	0.628
clutchsize	1	1	26	3.781299	0.0627
total_mov	1	1	26	1.0736779	0.3097
total_mov*repeat	1	1	26	0.0019814	0.9648
clutchsize*repeat	1	1	26	0.8085183	0.3768

Fit Mixed

Actual by Predicted Plot

Fit Statistics

-2 Residual Log Likelihood	820.86956
-2 Log Likelihood	832.01928
AICc	857.53928
BIC	881.14427

Repeated Effects Covariance Parameter Estimates
Repeated Effect: repeat2

Covariance Parameter	Estimate	Std Error	95% Lower	95% Upper
Var(1)	1806.5626	506.99136	812.87782	2800.2474
Cov(2,1)	48.289843	134.48275	-215.2915	311.87118
Var(2)	259.4921	73.871429	114.70676	404.27744
Cov(3,1)	259.44369	325.58064	-378.6826	897.57003
Cov(3,2)	0.2462116	125.81782	-246.3522	246.84461
Var(3)	1511.9761	428.89932	671.34892	2352.6033

Fixed Effects Parameter Estimates

Term	Estimate	Std Error	DFDen	t Ratio	Prob>|t|	95% Lower	95% Upper
Intercept	42.302152	18.786194	45.1	2.25	0.0293	4.4674046	80.136899
repeat	-2.996176	5.1106745	27	-0.59	0.5626	-13.48241	7.4900591
clutchsize	-3.451698	1.7571319	26	-1.96	0.0603	-7.063512	0.1601151
total_mov	0.2874947	0.2746865	26	1.05	0.3049	-0.277122	0.8521112
(total_mov-22.6552)*(repeat-2)	-0.082695	0.4949015	27	-0.17	0.8685	-1.098149	0.9327585

Fixed Effects Tests

Source	Nparm	DFNum	DFDen	F Ratio	Prob > F
repeat	1	1	27	0.3436989	0.5626
clutchsize	1	1	26	3.8588418	0.0603
total_mov	1	1	26	1.0954315	0.3049
total_mov*repeat	1	1	27	0.0279205	0.8685

Fit Mixed

Actual by Predicted Plot

Fit Statistics

-2 Residual Log Likelihood	816.36667
-2 Log Likelihood	831.10549
AICc	856.62549

BIC	880.23048

Repeated Effects Covariance Parameter Estimates
Repeated Effect: repeat2

Covariance Parameter	Estimate	Std Error	95% Lower	95% Upper
Var(1)	1859.6465	523.78801	833.04083	2886.2521
Cov(2,1)	40.035053	137.32533	-229.1177	309.18776
Var(2)	258.60675	73.389612	114.76575	402.44774
Cov(3,1)	279.19374	322.51528	-352.9246	911.31207
Cov(3,2)	10.889457	120.30726	-224.9084	246.68735
Var(3)	1432.4717	403.91979	640.80345	2224.14

Fixed Effects Parameter Estimates

Term	Estimate	Std Error	DFDen	t Ratio	Prob>|t|	95% Lower	95% Upper
Intercept	40.233597	18.425269	43.5	2.18	0.0344	3.0888579	77.378337
repeat	-2.496816	5.0318226	27	-0.5	0.6238	-12.82126	7.8276311
clutchsize	-3.314907	1.7053059	26	-1.94	0.0628	-6.820074	0.19026
total_mov	0.2834638	0.2826693	26	1	0.3252	-0.297567	0.8644947
(clutchsize-8.31034)*(repeat-2)	-2.807535	3.0256485	27	-0.93	0.3617	-9.015653	3.4005823

Fixed Effects Tests

Source	Nparm	DFNum	DFDen	F Ratio	Prob > F
repeat	1	1	27	0.2462194	0.6238
clutchsize	1	1	26	3.7786629	0.0628
total_mov	1	1	26	1.0056296	0.3252
clutchsize*repeat	1	1	27	0.8610205	0.3617

Fit Mixed

Actual by Predicted Plot

Fit Statistics

-2 Residual Log Likelihood	816.63927
-2 Log Likelihood	832.22779
AICc	855.12253
BIC	876.88687

Repeated Effects Covariance Parameter Estimates
Repeated Effect: repeat2

Covariance Parameter	Estimate	Std Error	95% Lower	95% Upper
Var(1)	1905.6277	534.69031	857.65393	2953.6014
Cov(2,1)	58.874999	135.91249	-207.5086	325.25859
Var(2)	250.3074	69.432145	114.2229	386.3919
Cov(3,1)	319.9465	329.01858	-324.9181	964.81106
Cov(3,2)	24.50074	119.05567	-208.8441	257.84557
Var(3)	1467.996	412.40192	659.70309	2276.2889

Fixed Effects Parameter Estimates

Term	Estimate	Std Error	DFDen	t Ratio	Prob>|t|	95% Lower	95% Upper
Intercept	44.388648	17.707726	45.6	2.51	0.0158	8.7355251	80.041771
repeat	-2.496819	5.0318231	27	-0.5	0.6238	-12.82127	7.8276289
clutchsize	-3.076726	1.6986411	27	-1.81	0.0812	-6.562028	0.4085759
(clutchsize-8.31034)*(repeat-2)	-2.807534	3.0256487	27	-0.93	0.3617	-9.015652	3.4005838

Fixed Effects Tests

Source	Nparm	DFNum	DFDen	F Ratio	Prob > F
repeat	1	1	27	0.24622	0.6238
clutchsize	1	1	27	3.2807599	0.0812
clutchsize*repeat	1	1	27	0.8610197	0.3617

Fit Mixed

Actual by Predicted Plot

Fit Statistics

-2 Residual Log Likelihood	827.64193
-2 Log Likelihood	836.19327
AICc	859.08801
BIC	880.85236

Repeated Effects Covariance Parameter Estimates
Repeated Effect: repeat2

Covariance Parameter	Estimate	Std Error	95% Lower	95% Upper
Var(1)	1862.8577	522.05718	839.64445	2886.071
Cov(2,1)	78.742304	138.17746	-192.0805	349.56515
Var(2)	263.06942	72.309788	121.34484	404.794
Cov(3,1)	365.14376	347.70927	-316.3539	1046.6414
Cov(3,2)	80.185081	130.63781	-175.8603	336.23049
Var(3)	1667.0874	467.63681	750.5361	2583.6387

Fixed Effects Parameter Estimates

Term	Estimate	Std Error	DFDen	t Ratio	Prob>|t|	95% Lower	95% Upper
Intercept	14.720297	12.508872	42.3	1.18	0.2459	-10.5191	39.959691
repeat	-2.995163	5.110469	27	-0.59	0.5627	-13.48097	7.4906452
total_mov	0.2121012	0.2866777	27.2	0.74	0.4657	-0.375923	0.800125
(total_mov-22.6552)*(repeat-2)	-0.082651	0.4948816	27	-0.17	0.8686	-1.098063	0.9327611

Fixed Effects Tests

Source	Nparm	DFNum	DFDen	F Ratio	Prob > F
repeat	1	1	27	0.3434942	0.5627
total_mov	1	1	27.2	0.547392	0.4657
total_mov*repeat	1	1	27	0.027893	0.8686

Fit Mixed

Actual by Predicted Plot

Fit Statistics

-2 Residual Log Likelihood	809.93225
-2 Log Likelihood	829.64979
AICc	855.16979
BIC	878.77478

Repeated Effects Covariance Parameter Estimates
Repeated Effect: repeat2

Covariance Parameter	Estimate	Std Error	95% Lower	95% Upper
Var(1)	2005.6757	567.48981	893.41611	3117.9353
Cov(2,1)	102.28581	141.66937	-175.381	379.95267
Var(2)	237.06762	66.477991	106.77316	367.36209
Cov(3,1)	348.78964	334.2526	-306.3334	1003.9127
Cov(3,2)	-3.310405	115.49438	-229.6752	223.05442
Var(3)	1425.6288	400.82354	640.02908	2211.2285

Fixed Effects Parameter Estimates

Term	Estimate	Std Error	DFDen	t Ratio	Prob>|t|	95% Lower	95% Upper
Intercept	41.538142	17.739168	42.5	2.34	0.024	5.7525975	77.323687
repeat	-2.496905	5.0318226	27	-0.5	0.6238	-12.82135	7.8275414
age	5.5395429	3.5885587	26	1.54	0.1348	-1.836794	12.91588
clutchsize	-3.889676	1.7702946	26.1	-2.2	0.0371	-7.527983	-0.251369
(clutchsize-8.31034)*(repeat-2)	-2.80751	3.0256484	27	-0.93	0.3617	-9.015627	3.4006077

Fixed Effects Tests

Source	Nparm	DFNum	DFDen	F Ratio	Prob > F
repeat	1	1	27	0.246237	0.6238
age	1	1	26	2.3829106	0.1348
clutchsize	1	1	26.1	4.8276496	0.0371
clutchsize*repeat	1	1	27	0.8610048	0.3617

Fit Mixed

Actual by Predicted Plot

Fit Statistics

-2 Residual Log Likelihood	822.95369
-2 Log Likelihood	835.9014
AICc	861.4214
BIC	885.02639

Repeated Effects Covariance Parameter Estimates
Repeated Effect: repeat2

Covariance Parameter	Estimate	Std Error	95% Lower	95% Upper
Var(1)	1918.287	544.35407	851.37261	2985.2013
Cov(2,1)	107.31898	147.85559	-182.4727	397.11061
Var(2)	265.77869	74.628427	119.50966	412.04772
Cov(3,1)	396.90716	357.76896	-304.3071	1098.1214
Cov(3,2)	84.976757	132.89915	-175.5008	345.45431
Var(3)	1675.1722	470.00128	753.98666	2596.3578

Fixed Effects Parameter Estimates

Term	Estimate	Std Error	DFDen	t Ratio	Prob>|t|	95% Lower	95% Upper
Intercept	11.652997	13.704567	46.1	0.85	0.3996	-15.93163	39.237621
repeat	-2.996372	5.1106718	27	-0.59	0.5625	-13.4826	7.4898569
age	2.1947475	3.7791318	26	0.58	0.5664	-5.573286	9.9627811
total_mov	0.1765903	0.2956259	26	0.6	0.5554	-0.43107	0.7842508
(total_mov-22.6552)*(repeat-2)	-0.082704	0.4949012	27	-0.17	0.8685	-1.098157	0.9327494

Fixed Effects Tests

Source	Nparm	DFNum	DFDen	F Ratio	Prob > F
repeat	1	1	27	0.3437444	0.5625
age	1	1	26	0.3372757	0.5664

total_mov	1	1	26	0.3568197	0.5554
total_mov*repeat	1	1	27	0.0279262	0.8685

Fit Mixed

Actual by Predicted Plot

Fit Statistics

-2 Residual Log Likelihood	809.93225
-2 Log Likelihood	829.64979
AICc	855.16979
BIC	878.77478

Repeated Effects Covariance Parameter Estimates
Repeated Effect: repeat2

Covariance Parameter	Estimate	Std Error	95% Lower	95% Upper
Var(1)	2005.6757	567.48981	893.41611	3117.9353
Cov(2,1)	102.28581	141.66937	-175.381	379.95267
Var(2)	237.06762	66.477991	106.77316	367.36209
Cov(3,1)	348.78964	334.2526	-306.3334	1003.9127
Cov(3,2)	-3.310405	115.49438	-229.6752	223.05442
Var(3)	1425.6288	400.82354	640.02908	2211.2285

Fixed Effects Parameter Estimates

Term	Estimate	Std Error	DFDen	t Ratio	Prob>|t|	95% Lower	95% Upper
Intercept	41.538142	17.739168	42.5	2.34	0.024	5.7525975	77.323687
repeat	-2.496905	5.0318226	27	-0.5	0.6238	-12.82135	7.8275414
age	5.5395429	3.5885587	26	1.54	0.1348	-1.836794	12.91588
clutchsize	-3.889676	1.7702946	26.1	-2.2	0.0371	-7.527983	-0.251369
(clutchsize-8.31034)*(repeat-2)	-2.80751	3.0256484	27	-0.93	0.3617	-9.015627	3.4006077

Fixed Effects Tests

Source	Nparm	DFNum	DFDen	F Ratio	Prob > F
repeat	1	1	27	0.246237	0.6238
age	1	1	26	2.3829106	0.1348
clutchsize	1	1	26.1	4.8276496	0.0371
clutchsize*repeat	1	1	27	0.8610048	0.3617

Fit Mixed

Actual by Predicted Plot

Fit Statistics

-2 Residual Log Likelihood	816.30072
-2 Log Likelihood	832.31975
AICc	857.83975
BIC	881.44474

Repeated Effects Covariance Parameter Estimates
Repeated Effect: repeat2

Covariance Parameter	Estimate	Std Error	95% Lower	95% Upper
Var(1)	1936.7428	556.49716	846.02843	3027.4572

Cov(2,1)	59.237816	137.2155	-209.6996	328.17526
Var(2)	250.30741	69.432148	114.2229	386.39192
Cov(3,1)	293.6465	335.29046	-363.5107	950.80373
Cov(3,2)	24.137915	120.14815	-211.3481	259.62396
Var(3)	1489.4808	429.59371	647.49263	2331.469

Fixed Effects Parameter Estimates

Term	Estimate	Std Error	DFDen	t Ratio	Prob>|t|	95% Lower	95% Upper
Intercept	44.340707	17.832614	45.5	2.49	0.0166	8.4355578	80.245855
repeat	-2.472848	5.1382535	26	-0.48	0.6344	-13.03471	8.0890156
clutchsize	-3.076726	1.6986411	27	-1.81	0.0812	-6.562028	0.4085759
(total_mov-22.6552)*(repeat-2)	0.0236339	0.5148897	26	0.05	0.9637	-1.034737	1.0820049
(clutchsize-8.31034)*(repeat-2)	-2.827392	3.1025906	26	-0.91	0.3705	-9.20484	3.5500553

Fixed Effects Tests

Source	Nparm	DFNum	DFDen	F Ratio	Prob > F
repeat	1	1	26	0.2316134	0.6344
clutchsize	1	1	27	3.2807599	0.0812
total_mov*repeat	1	1	26	0.0021069	0.9637
clutchsize*repeat	1	1	26	0.8304687	0.3705

Fit Mixed

Actual by Predicted Plot

Fit Statistics

-2 Residual Log Likelihood	821.17247
-2 Log Likelihood	833.16665
AICc	856.06139
BIC	877.82574

Repeated Effects Covariance Parameter Estimates
Repeated Effect: repeat2

Covariance Parameter	Estimate	Std Error	95% Lower	95% Upper
Var(1)	1852.0092	522.13623	828.64099	2875.3774
Cov(2,1)	63.335554	132.52435	-196.4074	323.07851
Var(2)	250.89413	69.751807	114.1831	387.60516
Cov(3,1)	300.43147	332.36013	-350.9824	951.84535
Cov(3,2)	17.894092	123.71251	-224.578	260.36616
Var(3)	1549.7216	441.91788	683.57844	2415.8647

Fixed Effects Parameter Estimates

Term	Estimate	Std Error	DFDen	t Ratio	Prob>|t|	95% Lower	95% Upper
Intercept	46.395466	18.14059	47.9	2.56	0.0138	9.9184412	82.87249
repeat	-2.958353	5.1110359	27	-0.58	0.5675	-13.44536	7.5286529
clutchsize	-3.204795	1.7512733	27	-1.83	0.0783	-6.798171	0.3885814
(total_mov-22.6552)*(repeat-2)	-0.040043	0.5102686	27	-0.08	0.938	-1.087044	1.0069583

Fixed Effects Tests

Source	Nparm	DFNum	DFDen	F Ratio	Prob > F
repeat	1	1	27	0.3350288	0.5675
clutchsize	1	1	27	3.3488264	0.0783
total_mov*repeat	1	1	27	0.0061582	0.938

Fit Mixed

Actual by Predicted Plot

Fit Statistics

-2 Residual Log Likelihood	822.59619
-2 Log Likelihood	835.152
AICc	860.672
BIC	884.27699

Repeated Effects Covariance Parameter Estimates
Repeated Effect: repeat2

Covariance Parameter	Estimate	Std Error	95% Lower	95% Upper
Var(1)	2017.0227	588.05794	864.45029	3169.5951
Cov(2,1)	101.87532	145.90083	-184.085	387.83569
Var(2)	263.06496	72.307339	121.34518	404.78474
Cov(3,1)	354.12401	349.24756	-330.3886	1038.6366
Cov(3,2)	57.046274	127.06575	-191.998	306.09058
Var(3)	1534.965	449.33513	654.28429	2415.6456

Fixed Effects Parameter Estimates

Term	Estimate	Std Error	DFDen	t Ratio	Prob>|t|	95% Lower	95% Upper
Intercept	13.583585	12.741377	38.7	1.07	0.293	-12.19391	39.361077
repeat	-2.426807	5.1414264	26	-0.47	0.6409	-12.9961	8.142486
total_mov	0.2121002	0.2866751	27.2	0.74	0.4657	-0.375917	0.8001178
(total_mov-22.6552)*(repeat-2)	-0.010587	0.4999773	26	-0.02	0.9833	-1.038388	1.0172145
(clutchsize-8.31034)*(repeat-2)	-3.323898	3.2082854	26	-1.04	0.3097	-9.918619	3.2708233

Fixed Effects Tests

Source	Nparm	DFNum	DFDen	F Ratio	Prob > F
repeat	1	1	26	0.2227939	0.6409
total_mov	1	1	27.2	0.5473969	0.4657
total_mov*repeat	1	1	26	0.0004484	0.9833
clutchsize*repeat	1	1	26	1.0733696	0.3097

Fit Mixed

Actual by Predicted Plot

Fit Statistics

-2 Residual Log Likelihood	822.94158
-2 Log Likelihood	835.05837
AICc	857.95311
BIC	879.71745

Repeated Effects Covariance Parameter Estimates
Repeated Effect: repeat2

Covariance Parameter	Estimate	Std Error	95% Lower	95% Upper
Var(1)	1989.358	569.30374	873.54312	3105.1728
Cov(2,1)	100.29625	143.81746	-181.5808	382.17329
Var(2)	262.96696	72.254756	121.35024	404.58368
Cov(3,1)	380.04512	343.32377	-292.8571	1052.9474
Cov(3,2)	58.54563	125.12111	-186.6872	303.7785
Var(3)	1510.8085	434.17184	659.84737	2361.7697

Fixed Effects Parameter Estimates

Term	Estimate	Std Error	DFDen	t Ratio	Prob>|t|	95% Lower	95% Upper
Intercept	13.580683	12.66871	41.7	1.07	0.2899	-11.99068	39.152049
repeat	-2.419024	5.0347322	27	-0.48	0.6348	-12.75023	7.9121856
total_mov	0.2115122	0.2958203	27.1	0.72	0.4807	-0.395326	0.8183501
(clutchsize-8.31034)*(repeat-2)	-3.312914	3.1248791	27	-1.06	0.2985	-9.724595	3.0987677

Fixed Effects Tests

Source	Nparm	DFNum	DFDen	F Ratio	Prob > F
repeat	1	1	27	0.2308489	0.6348
total_mov	1	1	27.1	0.5112284	0.4807
clutchsize*repeat	1	1	27	1.1239678	0.2985

Fit Mixed

Actual by Predicted Plot

Fit Statistics

-2 Residual Log Likelihood	822.4257
-2 Log Likelihood	835.71212
AICc	858.60686
BIC	880.3712

Repeated Effects Covariance Parameter Estimates
Repeated Effect: repeat2

Covariance Parameter	Estimate	Std Error	95% Lower	95% Upper
Var(1)	2046.7429	597.70554	875.26156	3218.2242
Cov(2,1)	109.78704	143.42306	-171.317	390.89107
Var(2)	255.79184	69.228319	120.10683	391.47686
Cov(3,1)	377.24193	352.38125	-313.4126	1067.8965
Cov(3,2)	64.2122	124.64274	-180.0831	308.50748
Var(3)	1552.8545	456.26541	658.59075	2447.1183

Fixed Effects Parameter Estimates

Term	Estimate	Std Error	DFDen	t Ratio	Prob>|t|	95% Lower	95% Upper
Intercept	18.154648	10.953823	25.5	1.66	0.1097	-4.381986	40.691282
repeat	-2.397598	5.1424821	26	-0.47	0.6449	-12.96891	8.1737134
(total_mov-22.6552)*(repeat-2)	0.0236342	0.5165938	26	0.05	0.9639	-1.038239	1.0855079
(clutchsize-8.31034)*(repeat-2)	-3.323353	3.2077595	26	-1.04	0.3097	-9.916997	3.2702911

Fixed Effects Tests

Source	Nparm	DFNum	DFDen	F Ratio	Prob > F
repeat	1	1	26	0.2173738	0.6449
total_mov*repeat	1	1	26	0.0020931	0.9639
clutchsize*repeat	1	1	26	1.0733697	0.3097

Fit Mixed

Actual by Predicted Plot

Fit Statistics

-2 Residual Log Likelihood	822.76426
-2 Log Likelihood	835.62016
AICc	855.95782
BIC	875.81333

Repeated Effects Covariance Parameter Estimates
Repeated Effect: repeat2

Covariance Parameter	Estimate	Std Error	95% Lower	95% Upper
Var(1)	2015.0655	573.89362	890.25472	3139.8764
Cov(2,1)	109.31181	141.96495	-168.9344	387.558
Var(2)	255.79188	69.22834	120.10683	391.47694
Cov(3,1)	403.60718	346.34977	-275.2259	1082.4402
Cov(3,2)	64.687454	123.4013	-177.1746	306.54955
Var(3)	1531.8013	437.73768	673.85125	2389.7514

Fixed Effects Parameter Estimates

Term	Estimate	Std Error	DFDen	t Ratio	Prob>|t|	95% Lower	95% Upper
Intercept	18.20578	10.745879	26.5	1.69	0.1019	-3.862375	40.273935
repeat	-2.423164	5.0345317	27	-0.48	0.6342	-12.75387	7.9075427
(clutchsize-8.31034)*(repeat-2)	-3.292995	3.1240143	27	-1.05	0.3012	-9.702943	3.1169526

Fixed Effects Tests

Source	Nparm	DFNum	DFDen	F Ratio	Prob > F
repeat	1	1	27	0.231658	0.6342
clutchsize*repeat	1	1	27	1.1111077	0.3012

Fit Mixed

Actual by Predicted Plot

Fit Statistics

-2 Residual Log Likelihood	827.47144
-2 Log Likelihood	836.75336
AICc	857.09102
BIC	876.94653

Repeated Effects Covariance Parameter Estimates
Repeated Effect: repeat2

Covariance Parameter	Estimate	Std Error	95% Lower	95% Upper
Var(1)	1888.3063	530.0324	849.46194	2927.1508
Cov(2,1)	86.808329	135.66451	-179.0892	352.70587
Var(2)	255.79199	69.228396	120.10682	391.47715
Cov(3,1)	388.56771	350.8008	-298.9892	1076.1247
Cov(3,2)	87.190984	128.19634	-164.0692	338.45119
Var(3)	1688.6394	474.74588	758.15455	2619.1242

Fixed Effects Parameter Estimates

Term	Estimate	Std Error	DFDen	t Ratio	Prob>|t|	95% Lower	95% Upper
Intercept	19.340475	10.685007	27.1	1.81	0.0814	-2.580623	41.261573
repeat	-2.990511	5.1108319	27	-0.59	0.5633	-13.47707	7.4960452
(total_mov-22.6552)*(repeat-2)	-0.076234	0.5111952	27	-0.15	0.8826	-1.125119	0.9726523

Fixed Effects Tests

Source	Nparm	DFNum	DFDen	F Ratio	Prob > F
repeat	1	1	27	0.3423794	0.5633
total_mov*repeat	1	1	27	0.0222392	0.8826

Strophes sung Fit Group

Fit Mixed

Actual by Predicted Plot

Fit Statistics

-2 Residual Log Likelihood	692.6744
-2 Log Likelihood	702.42256
AICc	733.40886
BIC	760.47937

Repeated Effects Covariance Parameter Estimates
Repeated Effect: repeat2

Covariance Parameter	Estimate	Std Error	95% Lower	95% Upper
Var(1)	227.25174	66.373469	97.162136	357.34135
Cov(2,1)	29.62183	38.461489	-45.7613	105.00496
Var(2)	161.44066	47.775941	67.80154	255.07979
Cov(3,1)	139.39357	64.918271	12.156096	266.63104
Cov(3,2)	48.555046	47.07942	-43.71892	140.82901
Var(3)	326.73437	98.429043	133.81699	519.65175

Fixed Effects Parameter Estimates

Term	Estimate	Std Error	DFDen	t Ratio	Prob>|t|	95% Lower	95% Upper
Intercept	31.398385	11.396601	29.1	2.76	0.01	8.0944809	54.702288
repeat	-2.270846	1.6011258	26	-1.42	0.168	-5.561995	1.020303
age	-7.460532	2.661229	25.1	-2.8	0.0096	-12.94055	-1.980509
clutchsize	1.5692942	1.3018285	25.2	1.21	0.2392	-1.110845	4.2494337
total_mov	0.3834239	0.2010785	25.1	1.91	0.0681	-0.030644	0.7974917
(total_mov-22.6552)*(repeat-2)	0.2070227	0.1556595	26	1.33	0.1951	-0.112939	0.5269841
(clutchsize-8.31034)*(repeat-2)	1.8289177	0.9665585	26	1.89	0.0697	-0.157864	3.8156998

Fixed Effects Tests

Source	Nparm	DFNum	DFDen	F Ratio	Prob > F
repeat	1	1	26	2.0115207	0.168
age	1	1	25.1	7.8591405	0.0096
clutchsize	1	1	25.2	1.4531192	0.2392
total_mov	1	1	25.1	3.6360269	0.0681
total_mov*repeat	1	1	26	1.7688258	0.1951
clutchsize*repeat	1	1	26	3.580404	0.0697

Fit Mixed

Actual by Predicted Plot

Fit Statistics

-2 Residual Log Likelihood	703.37785
-2 Log Likelihood	710.20007
AICc	738.41629
BIC	763.79097

Repeated Effects Covariance Parameter Estimates
Repeated Effect: repeat2

Covariance Parameter	Estimate	Std Error	95% Lower	95% Upper
Var(1)	240.66343	68.79827	105.8213	375.50556
Cov(2,1)	66.854432	47.368316	-25.98576	159.69463
Var(2)	221.99525	64.049903	96.459748	347.53075
Cov(3,1)	125.26891	58.91079	9.8058825	240.73194
Cov(3,2)	58.080897	50.497616	-40.89261	157.05441
Var(3)	284.90589	81.825083	124.53167	445.28011

Fixed Effects Parameter Estimates

Term	Estimate	Std Error	DFDen	t Ratio	Prob>|t|	95% Lower	95% Upper
Intercept	30.91317	12.532188	29.6	2.47	0.0197	5.3038586	56.522481
repeat	-2.269486	1.6004215	26	-1.42	0.168	-5.558964	1.0199923
clutchsize	0.4380229	1.3596441	26.1	0.32	0.7499	-2.356262	3.2323078
total_mov	0.2276517	0.2189639	26.1	1.04	0.308	-0.222354	0.6776575
(total_mov-22.6552)*(repeat-2)	0.2071506	0.155591	26	1.33	0.1946	-0.112648	0.5269496
(clutchsize-8.31034)*(repeat-2)	1.8288928	0.9661333	26	1.89	0.0695	-0.156881	3.8146663

Fixed Effects Tests

Source	Nparm	DFNum	DFDen	F Ratio	Prob > F
repeat	1	1	26	2.0108804	0.168
clutchsize	1	1	26.1	0.103787	0.7499
total_mov	1	1	26.1	1.0809277	0.308
total_mov*repeat	1	1	26	1.7725704	0.1946
clutchsize*repeat	1	1	26	3.5834585	0.0695

Fit Mixed

Actual by Predicted Plot

Fit Statistics

-2 Residual Log Likelihood	708.82597
-2 Log Likelihood	714.1731
AICc	739.6931
BIC	763.29809

Repeated Effects Covariance Parameter Estimates
Repeated Effect: repeat2

Covariance Parameter	Estimate	Std Error	95% Lower	95% Upper
Var(1)	246.55536	71.290818	106.82792	386.2828
Cov(2,1)	66.87325	48.308117	-27.80892	161.55542
Var(2)	221.99647	64.052334	96.456198	347.53673
Cov(3,1)	117.89388	59.45752	1.3592813	234.42848
Cov(3,2)	58.144968	51.789323	-43.36024	159.65018
Var(3)	293.85717	85.424651	126.42793	461.28641

Fixed Effects Parameter Estimates

Term	Estimate	Std Error	DFDen	t Ratio	Prob>|t|	95% Lower	95% Upper
Intercept	31.755663	12.924337	29.8	2.46	0.0201	5.3514921	58.159833
repeat	-2.25337	1.6843586	27.1	-1.34	0.1921	-5.709082	1.2023428

clutchsize	0.3284827	1.4102289	26.1	0.23	0.8176	-2.569794	3.2267596
total_mov	0.2292306	0.2190499	26.1	1.05	0.3049	-0.22096	0.6794216
(total_mov-22.6552)*(repeat-2)	0.2347436	0.1631079	27.1	1.44	0.1616	-0.099897	0.5693838

Fixed Effects Tests

Source	Nparm	DFNum	DFDen	F Ratio	Prob > F
repeat	1	1	27.1	1.789764	0.1921
clutchsize	1	1	26.1	0.0542558	0.8176
total_mov	1	1	26.1	1.0951129	0.3049
total_mov*repeat	1	1	27.1	2.0712727	0.1616

Fit Mixed

Actual by Predicted Plot

Fit Statistics

-2 Residual Log Likelihood	703.04882
-2 Log Likelihood	711.89227
AICc	737.41227
BIC	761.01726

Repeated Effects Covariance Parameter Estimates
Repeated Effect: repeat2

Covariance Parameter	Estimate	Std Error	95% Lower	95% Upper
Var(1)	253.83091	74.134741	108.52949	399.13233
Cov(2,1)	58.537195	47.621644	-34.79951	151.8739
Var(2)	223.18308	64.760339	96.255151	350.11102
Cov(3,1)	123.63637	58.750933	8.4866602	238.78609
Cov(3,2)	66.523604	49.969537	-31.41489	164.4621
Var(3)	271.86164	75.314728	124.24748	419.47579

Fixed Effects Parameter Estimates

Term	Estimate	Std Error	DFDen	t Ratio	Prob>|t|	95% Lower	95% Upper
Intercept	31.775902	12.586244	29.6	2.52	0.0172	6.0586312	57.493172
repeat	-2.592336	1.609819	27	-1.61	0.1189	-5.895314	0.7106421
clutchsize	0.4443809	1.3607761	26.1	0.33	0.7466	-2.352296	3.2410573
total_mov	0.2148118	0.2266021	26.1	0.95	0.3518	-0.250901	0.680525
(clutchsize-8.31034)*(repeat-2)	1.9320135	0.9679885	27	2	0.0561	-0.054076	3.9181032

Fixed Effects Tests

Source	Nparm	DFNum	DFDen	F Ratio	Prob > F
repeat	1	1	27	2.5931553	0.1189
clutchsize	1	1	26.1	0.1066442	0.7466
total_mov	1	1	26.1	0.8986452	0.3518
clutchsize*repeat	1	1	27	3.9836386	0.0561

Fit Mixed

Actual by Predicted Plot

Fit Statistics

-2 Residual Log Likelihood	702.60222

-2 Log Likelihood	712.72837
AICc	735.62311
BIC	757.38745

Repeated Effects Covariance Parameter Estimates
Repeated Effect: repeat2

Covariance Parameter	Estimate	Std Error	95% Lower	95% Upper
Var(1)	232.43922	63.943478	107.1123	357.76613
Cov(2,1)	56.788292	46.887749	-35.11001	148.68659
Var(2)	241.53511	66.546159	111.10703	371.96319
Cov(3,1)	110.7243	53.07422	6.7007441	214.74786
Cov(3,2)	73.472979	50.949581	-26.38637	173.33232
Var(3)	267.51596	73.532435	123.39504	411.63689

Fixed Effects Parameter Estimates

Term	Estimate	Std Error	DFDen	t Ratio	Prob>|t|	95% Lower	95% Upper
Intercept	35.421391	11.809737	31.1	3	0.0053	11.339447	59.503335
repeat	-2.594237	1.6101466	27	-1.61	0.1188	-5.897985	0.7095107
clutchsize	0.551248	1.3472892	27	0.41	0.6857	-2.213139	3.3156354
(clutchsize-8.31034)*(repeat-2)	1.93195	0.9681855	27	2	0.0562	-0.054602	3.9185025

Fixed Effects Tests

Source	Nparm	DFNum	DFDen	F Ratio	Prob > F
repeat	1	1	27	2.5959034	0.1188
clutchsize	1	1	27	0.1674065	0.6857
clutchsize*repeat	1	1	27	3.981756	0.0562

Fit Mixed

Actual by Predicted Plot

Fit Statistics

-2 Residual Log Likelihood	711.2264
-2 Log Likelihood	714.14615
AICc	737.04088
BIC	758.80523

Repeated Effects Covariance Parameter Estimates
Repeated Effect: repeat2

Covariance Parameter	Estimate	Std Error	95% Lower	95% Upper
Var(1)	238.80809	67.520312	106.47071	371.14547
Cov(2,1)	60.627951	45.961767	-29.45546	150.71136
Var(2)	217.88843	62.629905	95.136075	340.64079
Cov(3,1)	113.80947	57.985994	0.1590063	227.45992
Cov(3,2)	55.655846	50.169679	-42.67492	153.98661
Var(3)	293.72275	83.682389	129.70828	457.73722

Fixed Effects Parameter Estimates

Term	Estimate	Std Error	DFDen	t Ratio	Prob>|t|	95% Lower	95% Upper
Intercept	34.377856	6.2433303	41.7	5.51	<.0001	21.775597	46.980115
repeat	-2.253564	1.6853887	27	-1.34	0.1923	-5.711694	1.2045654
total_mov	0.2340712	0.2147259	27	1.09	0.2853	-0.206509	0.6746517
(total_mov-22.6552)*(repeat-2)	0.2347253	0.1632077	27	1.44	0.1619	-0.100149	0.5695996

Fixed Effects Tests

Source	Nparm	DFNum	DFDen	F Ratio	Prob > F
repeat	1	1	27	1.7878856	0.1923
total_mov	1	1	27	1.1883028	0.2853
total_mov*repeat	1	1	27	2.0684201	0.1619

Fit Mixed

Actual by Predicted Plot

Fit Statistics

-2 Residual Log Likelihood	693.28248
-2 Log Likelihood	706.72559
AICc	732.24559
BIC	755.85058

Repeated Effects Covariance Parameter Estimates
Repeated Effect: repeat2

Covariance Parameter	Estimate	Std Error	95% Lower	95% Upper
Var(1)	211.20497	58.575903	96.398305	326.01163
Cov(2,1)	25.260149	40.210084	-53.55017	104.07047
Var(2)	199.73309	55.79294	90.380933	309.08524
Cov(3,1)	115.14059	55.395704	6.567007	223.71418
Cov(3,2)	67.597988	49.590774	-29.59814	164.79412
Var(3)	297.58002	85.173691	130.64266	464.51739

Fixed Effects Parameter Estimates

Term	Estimate	Std Error	DFDen	t Ratio	Prob>|t|	95% Lower	95% Upper
Intercept	38.18948	11.131023	30.3	3.43	0.0018	15.466694	60.912266
repeat	-2.594281	1.6101415	27	-1.61	0.1188	-5.898015	0.7094534
age	-6.612107	2.6881686	26	-2.46	0.0209	-12.13769	-1.086521
clutchsize	1.622547	1.3399413	26.1	1.21	0.2368	-1.131033	4.376127
(clutchsize-8.31034)*(repeat-2)	1.9319486	0.9681824	27	2	0.0562	-0.054596	3.9184929

Fixed Effects Tests

Source	Nparm	DFNum	DFDen	F Ratio	Prob > F
repeat	1	1	27	2.5960074	0.1188
age	1	1	26	6.0501583	0.0209
clutchsize	1	1	26.1	1.4663006	0.2368
clutchsize*repeat	1	1	27	3.9817755	0.0562

Fit Mixed

Actual by Predicted Plot

Fit Statistics

-2 Residual Log Likelihood	701.15157
-2 Log Likelihood	707.1779
AICc	732.6979
BIC	756.30289

Repeated Effects Covariance Parameter Estimates
Repeated Effect: repeat2

Covariance Parameter	Estimate	Std Error	95% Lower	95% Upper
Var(1)	220.62574	63.766502	95.645688	345.60578
Cov(2,1)	25.455523	37.907419	-48.84165	99.752699
Var(2)	165.73083	48.406799	70.855249	260.60642
Cov(3,1)	131.78189	64.984545	4.4145233	259.14926
Cov(3,2)	56.64129	49.452447	-40.28372	153.56631
Var(3)	347.85133	104.89229	142.26623	553.43644

Fixed Effects Parameter Estimates

Term	Estimate	Std Error	DFDen	t Ratio	Prob>|t|	95% Lower	95% Upper
Intercept	43.188052	6.7301294	39.2	6.42	<.0001	29.577824	56.798281
repeat	-2.253593	1.6853953	27	-1.34	0.1923	-5.711738	1.2045524
age	-6.913238	2.5250003	26	-2.74	0.011	-12.10344	-1.723038
total_mov	0.3941886	0.2029716	25.9	1.94	0.0631	-0.023143	0.8115198
(total_mov-22.6552)*(repeat-2)	0.2347227	0.1632083	27	1.44	0.1619	-0.100153	0.5695984

Fixed Effects Tests

Source	Nparm	DFNum	DFDen	F Ratio	Prob > F
repeat	1	1	27	1.787917	0.1923
age	1	1	26	7.4961814	0.011
total_mov	1	1	25.9	3.7717027	0.0631
total_mov*repeat	1	1	27	2.0683569	0.1619

Fit Mixed

Actual by Predicted Plot

Fit Statistics

-2 Residual Log Likelihood	693.28248
-2 Log Likelihood	706.72559
AICc	732.24559
BIC	755.85058

Repeated Effects Covariance Parameter Estimates
Repeated Effect: repeat2

Covariance Parameter	Estimate	Std Error	95% Lower	95% Upper
Var(1)	211.20497	58.575903	96.398305	326.01163
Cov(2,1)	25.260149	40.210084	-53.55017	104.07047
Var(2)	199.73309	55.79294	90.380933	309.08524
Cov(3,1)	115.14059	55.395704	6.567007	223.71418
Cov(3,2)	67.597988	49.590774	-29.59814	164.79412
Var(3)	297.58002	85.173691	130.64266	464.51739

Fixed Effects Parameter Estimates

Term	Estimate	Std Error	DFDen	t Ratio	Prob>|t|	95% Lower	95% Upper
Intercept	38.18948	11.131023	30.3	3.43	0.0018	15.466694	60.912266
repeat	-2.594281	1.6101415	27	-1.61	0.1188	-5.898015	0.7094534
age	-6.612107	2.6881686	26	-2.46	0.0209	-12.13769	-1.086521
clutchsize	1.622547	1.3399413	26.1	1.21	0.2368	-1.131033	4.376127
(clutchsize-8.31034)*(repeat-2)	1.9319486	0.9681824	27	2	0.0562	-0.054596	3.9184929

Fixed Effects Tests

Source	Nparm	DFNum	DFDen	F Ratio	Prob > F

repeat	1	1	27	2.5960074	0.1188
age	1	1	26	6.0501583	0.0209
clutchsize	1	1	26.1	1.4663006	0.2368
clutchsize*repeat	1	1	27	3.9817755	0.0562

Fit Mixed

Actual by Predicted Plot

Fit Statistics

-2 Residual Log Likelihood	703.09489
-2 Log Likelihood	711.2099
AICc	736.7299
BIC	760.33489

Repeated Effects Covariance Parameter Estimates
Repeated Effect: repeat2

Covariance Parameter	Estimate	Std Error	95% Lower	95% Upper
Var(1)	228.92156	62.982678	105.47778	352.36534
Cov(2,1)	69.993651	48.502665	-25.06983	165.05713
Var(2)	241.42931	66.488855	111.11355	371.74507
Cov(3,1)	111.64089	53.08657	7.5931266	215.68866
Cov(3,2)	60.266514	51.467776	-40.60847	161.1415
Var(3)	269.21464	74.247968	123.69129	414.73798

Fixed Effects Parameter Estimates

Term	Estimate	Std Error	DFDen	t Ratio	Prob>|t|	95% Lower	95% Upper
Intercept	34.795619	11.831883	31	2.94	0.0061	10.664726	58.926512
repeat	-2.281008	1.6010222	26	-1.42	0.1661	-5.571768	1.0097528
clutchsize	0.5512798	1.3471884	27	0.41	0.6856	-2.212871	3.3154307
(total_mov-22.6552)*(repeat-2)	0.2000642	0.1608745	26	1.24	0.2247	-0.1306	0.5307282
(clutchsize-8.31034)*(repeat-2)	1.8324045	0.9667546	26	1.9	0.0692	-0.154672	3.8194808

Fixed Effects Tests

Source	Nparm	DFNum	DFDen	F Ratio	Prob > F
repeat	1	1	26	2.0298258	0.1661
clutchsize	1	1	27	0.1674509	0.6856
total_mov*repeat	1	1	26	1.5465522	0.2247
clutchsize*repeat	1	1	26	3.5926108	0.0692

Fit Mixed

Actual by Predicted Plot

Fit Statistics

-2 Residual Log Likelihood	708.55697
-2 Log Likelihood	715.19843
AICc	738.09317
BIC	759.85751

Repeated Effects Covariance Parameter Estimates
Repeated Effect: repeat2

Covariance Parameter	Estimate	Std Error	95% Lower	95% Upper
Var(1)	236.17069	66.055911	106.70348	365.63789
Cov(2,1)	70.801928	49.749039	-26.7044	168.30825
Var(2)	241.5329	66.536289	111.12417	371.94163
Cov(3,1)	104.19331	53.691578	-1.040245	209.42687
Cov(3,2)	59.577186	52.814932	-43.93818	163.09255
Var(3)	276.70524	77.107478	125.57736	427.83312

Fixed Effects Parameter Estimates

Term	Estimate	Std Error	DFDen	t Ratio	Prob>|t|	95% Lower	95% Upper
Intercept	35.544798	12.279251	31.2	2.89	0.0069	10.50601	60.583587
repeat	-2.263477	1.6851093	27	-1.34	0.1904	-5.720798	1.1938444
clutchsize	0.4567107	1.39805	27	0.33	0.7464	-2.411837	3.3252584
(total_mov-22.6552)*(repeat-2)	0.2281712	0.169239	27	1.35	0.1888	-0.119062	0.5754046

Fixed Effects Tests

Source	Nparm	DFNum	DFDen	F Ratio	Prob > F
repeat	1	1	27	1.8042469	0.1904
clutchsize	1	1	27	0.1067178	0.7464
total_mov*repeat	1	1	27	1.8176935	0.1888

Fit Mixed

Actual by Predicted Plot

Fit Statistics

-2 Residual Log Likelihood	705.84114
-2 Log Likelihood	710.23895
AICc	735.75895
BIC	759.36394

Repeated Effects Covariance Parameter Estimates
Repeated Effect: repeat2

Covariance Parameter	Estimate	Std Error	95% Lower	95% Upper
Var(1)	236.77073	67.132799	105.19286	368.3486
Cov(2,1)	62.545055	45.826201	-27.27265	152.36276
Var(2)	217.88848	62.629935	95.136059	340.64089
Cov(3,1)	121.23703	57.502475	8.5342456	233.93981
Cov(3,2)	53.738636	48.996655	-42.29304	149.77031
Var(3)	280.90578	80.234075	123.64988	438.16168

Fixed Effects Parameter Estimates

Term	Estimate	Std Error	DFDen	t Ratio	Prob>|t|	95% Lower	95% Upper
Intercept	34.411419	6.1826511	40.2	5.57	<.0001	21.917858	46.904979
repeat	-2.270347	1.6011473	26	-1.42	0.1681	-5.561531	1.0208372
total_mov	0.2340714	0.214726	27	1.09	0.2853	-0.206509	0.674652
(total_mov-22.6552)*(repeat-2)	0.2072638	0.1557019	26	1.33	0.1947	-0.112783	0.527311
(clutchsize-8.31034)*(repeat-2)	1.8152961	0.9979818	26	1.82	0.0805	-0.236084	3.8666762

Fixed Effects Tests

Source	Nparm	DFNum	DFDen	F Ratio	Prob > F
repeat	1	1	26	2.0105825	0.1681
total_mov	1	1	27	1.1883042	0.2853
total_mov*repeat	1	1	26	1.7719817	0.1947
clutchsize*repeat	1	1	26	3.3086412	0.0805

Fit Mixed

Actual by Predicted Plot

Fit Statistics

-2 Residual Log Likelihood	705.5156
-2 Log Likelihood	711.92757
AICc	734.8223
BIC	756.58665

Repeated Effects Covariance Parameter Estimates
Repeated Effect: repeat2

Covariance Parameter	Estimate	Std Error	95% Lower	95% Upper
Var(1)	250.06858	72.309167	108.34522	391.79195
Cov(2,1)	54.328	45.961987	-35.75584	144.41184
Var(2)	218.30669	62.887081	95.050274	341.5631
Cov(3,1)	119.34502	57.156669	7.3200119	231.37004
Cov(3,2)	61.710567	48.106275	-32.576	155.99713
Var(3)	267.00176	73.282553	123.3706	410.63293

Fixed Effects Parameter Estimates

Term	Estimate	Std Error	DFDen	t Ratio	Prob>|t|	95% Lower	95% Upper
Intercept	35.301247	6.3148535	39.6	5.59	<.0001	22.534197	48.068297
repeat	-2.591735	1.6096665	27	-1.61	0.119	-5.894357	0.7108866
total_mov	0.2224238	0.2215827	27.1	1	0.3243	-0.232124	0.6769721
(clutchsize-8.31034)*(repeat-2)	1.9185265	1.0002225	27	1.92	0.0657	-0.133704	3.9707568

Fixed Effects Tests

Source	Nparm	DFNum	DFDen	F Ratio	Prob > F
repeat	1	1	27	2.5924443	0.119
total_mov	1	1	27.1	1.0076064	0.3243
clutchsize*repeat	1	1	27	3.6791062	0.0657

Fit Mixed

Actual by Predicted Plot

Fit Statistics

-2 Residual Log Likelihood	705.62069
-2 Log Likelihood	711.34852
AICc	734.24325
BIC	756.0076

Repeated Effects Covariance Parameter Estimates
Repeated Effect: repeat2

Covariance Parameter	Estimate	Std Error	95% Lower	95% Upper
Var(1)	224.96114	61.262507	104.88883	345.03344
Cov(2,1)	66.25959	47.069048	-25.99405	158.51323
Var(2)	237.89133	65.136012	110.22709	365.55557
Cov(3,1)	107.39805	51.554395	6.3532894	208.44281
Cov(3,2)	56.231977	49.970273	-41.70796	154.17191
Var(3)	264.65689	72.343238	122.86674	406.44703

Fixed Effects Parameter Estimates

Term	Estimate	Std Error	DFDen	t Ratio	Prob>|t|	95% Lower	95% Upper
Intercept	39.369118	3.8108613	26.5	10.33	<.0001	31.543034	47.195201
repeat	-2.280966	1.6009322	26	-1.42	0.1661	-5.571503	1.0095716
(total_mov-22.6552)*(repeat-2)	0.2001035	0.1607149	26	1.25	0.2242	-0.130231	0.5304376
(clutchsize-8.31034)*(repeat-2)	1.8153788	0.99795	26	1.82	0.0804	-0.235812	3.8665693

Fixed Effects Tests

Source	Nparm	DFNum	DFDen	F Ratio	Prob > F
repeat	1	1	26	2.0299793	0.1661
total_mov*repeat	1	1	26	1.5502339	0.2242
clutchsize*repeat	1	1	26	3.3091538	0.0804

Fit Mixed

Actual by Predicted Plot

Fit Statistics

-2 Residual Log Likelihood	705.12801
-2 Log Likelihood	712.86706
AICc	733.20473
BIC	753.06024

Repeated Effects Covariance Parameter Estimates
Repeated Effect: repeat2

Covariance Parameter	Estimate	Std Error	95% Lower	95% Upper
Var(1)	228.68581	62.33203	106.51727	350.85434
Cov(2,1)	53.156177	45.515141	-36.05186	142.36421
Var(2)	238.01562	65.203183	110.21972	365.81151
Cov(3,1)	106.48737	51.542833	5.4652708	207.50946
Cov(3,2)	69.35537	49.363189	-27.3947	166.10544
Var(3)	262.72709	71.48069	122.62751	402.82667

Fixed Effects Parameter Estimates

Term	Estimate	Std Error	DFDen	t Ratio	Prob>|t|	95% Lower	95% Upper
Intercept	39.995166	3.7412557	27.4	10.69	<.0001	32.323784	47.666549
repeat	-2.594584	1.6099436	27	-1.61	0.1187	-5.897842	0.7086735
(clutchsize-8.31034)*(repeat-2)	1.8967895	0.9959379	27	1.9	0.0675	-0.146703	3.9402824

Fixed Effects Tests

Source	Nparm	DFNum	DFDen	F Ratio	Prob > F
repeat	1	1	27	2.5972531	0.1187
clutchsize*repeat	1	1	27	3.6272189	0.0675

Fit Mixed

Actual by Predicted Plot

Fit Statistics

-2 Residual Log Likelihood	711.00595
-2 Log Likelihood	715.25642
AICc	735.59408
BIC	755.44959

Repeated Effects Covariance Parameter Estimates
Repeated Effect: repeat2

Covariance Parameter	Estimate	Std Error	95% Lower	95% Upper
Var(1)	227.28167	61.745177	106.26335	348.29999
Cov(2,1)	64.207424	47.325418	-28.54869	156.96354
Var(2)	237.90146	65.141546	110.22637	365.57654
Cov(3,1)	100.01087	52.108208	-2.119338	202.14108
Cov(3,2)	58.310997	51.246581	-42.13046	158.75245
Var(3)	277.11086	75.568011	129.00028	425.22144

Fixed Effects Parameter Estimates

Term	Estimate	Std Error	DFDen	t Ratio	Prob>|t|	95% Lower	95% Upper
Intercept	39.348736	3.9089603	27	10.07	<.0001	31.328765	47.368706
repeat	-2.270848	1.6851408	27	-1.35	0.189	-5.728263	1.1865666
(total_mov-22.6552)*(repeat-2)	0.2233567	0.1682886	27	1.33	0.1955	-0.121923	0.5686364

Fixed Effects Tests

Source	Nparm	DFNum	DFDen	F Ratio	Prob > F
repeat	1	1	27	1.8159498	0.189
total_mov*repeat	1	1	27	1.7615237	0.1955

Locomotor behavior Fit Group

Fit Mixed

Actual by Predicted Plot

Fit Statistics

-2 Residual Log Likelihood	596.08099
-2 Log Likelihood	599.51583
AICc	630.50214
BIC	657.57264

Repeated Effects Covariance Parameter Estimates
Repeated Effect: repeat2

Covariance Parameter	Estimate	Std Error	95% Lower	95% Upper
Var(1)	114.84293	32.261224	51.612095	178.07377
Cov(2,1)	64.928125	24.486693	16.93509	112.92116
Var(2)	92.233452	25.848324	41.571667	142.89524
Cov(3,1)	52.298937	19.983478	13.13204	91.465834
Cov(3,2)	42.151609	17.533256	7.7870587	76.51616
Var(3)	64.77174	18.328875	28.847805	100.69567

Fixed Effects Parameter Estimates

Term	Estimate	Std Error	DFDen	t Ratio	Prob>|t|	95% Lower	95% Upper
Intercept	22.696378	8.6772142	26.4	2.62	0.0145	4.8716964	40.52106
repeat	-4.791718	0.83218	26	-5.76	<.0001	-6.502288	-3.081147
age	2.2866762	1.9363269	25	1.18	0.2487	-1.701263	6.2746153

clutchsize	-0.33522	0.9868971	25.3	-0.34	0.7369	-2.366523	1.6960825
total_mov	-0.032203	0.1529569	24.9	-0.21	0.835	-0.34728	0.2828737
(total_mov-22.6552)*(repeat-2)	0.1557727	0.0809035	26	1.93	0.0652	-0.010527	0.3220723
(clutchsize-8.31034)*(repeat-2)	0.2224968	0.5023657	26	0.44	0.6615	-0.810131	1.2551242

Fixed Effects Tests

Source	Nparm	DFNum	DFDen	F Ratio	Prob > F
repeat	1	1	26	33.154908	<.0001
age	1	1	25	1.3946075	0.2487
clutchsize	1	1	25.3	0.1153763	0.7369
total_mov	1	1	24.9	0.0443265	0.835
total_mov*repeat	1	1	26	3.7072184	0.0652
clutchsize*repeat	1	1	26	0.1961587	0.6615

Fit Mixed

Actual by Predicted Plot

Fit Statistics

-2 Residual Log Likelihood	600.53958
-2 Log Likelihood	601.01261
AICc	629.22882
BIC	654.60351

Repeated Effects Covariance Parameter Estimates
Repeated Effect: repeat2

Covariance Parameter	Estimate	Std Error	95% Lower	95% Upper
Var(1)	121.9372	33.738306	55.811335	188.06306
Cov(2,1)	72.211274	25.741242	21.759365	122.66318
Var(2)	99.701916	27.15778	46.473645	152.93019
Cov(3,1)	55.061508	20.253855	15.364681	94.758334
Cov(3,2)	45.102624	17.82113	10.17385	80.031398
Var(3)	63.201839	17.468281	28.964636	97.439041

Fixed Effects Parameter Estimates

Term	Estimate	Std Error	DFDen	t Ratio	Prob>|t|	95% Lower	95% Upper
Intercept	22.956282	8.9338727	27.5	2.57	0.0159	4.6396995	41.272864
repeat	-4.791717	0.8321738	26	-5.76	<.0001	-6.502273	-3.081162
clutchsize	0.0145424	0.9692444	26	0.02	0.9881	-1.977762	2.006847
total_mov	-0.001304	0.156092	26	-0.01	0.9934	-0.322155	0.3195469
(total_mov-22.6552)*(repeat-2)	0.1557728	0.0809029	26	1.93	0.0652	-0.010525	0.322071
(clutchsize-8.31034)*(repeat-2)	0.2224967	0.5023619	26	0.44	0.6615	-0.810122	1.2551154

Fixed Effects Tests

Source	Nparm	DFNum	DFDen	F Ratio	Prob > F
repeat	1	1	26	33.155404	<.0001
clutchsize	1	1	26	0.0002251	0.9881
total_mov	1	1	26	6.98E-05	0.9934
total_mov*repeat	1	1	26	3.7072795	0.0652
clutchsize*repeat	1	1	26	0.1961616	0.6615

Fit Mixed

Actual by Predicted Plot

Fit Statistics

-2 Residual Log Likelihood	601.12945
-2 Log Likelihood	601.15195
AICc	626.67195
BIC	650.27694

Repeated Effects Covariance Parameter Estimates
Repeated Effect: repeat2

Covariance Parameter	Estimate	Std Error	95% Lower	95% Upper
Var(1)	120.25019	32.907757	55.752171	184.74821
Cov(2,1)	71.444142	25.315213	21.827236	121.06105
Var(2)	99.357021	27.000928	46.436174	152.27787
Cov(3,1)	55.29729	20.10265	15.89682	94.69776
Cov(3,2)	45.210546	17.795026	10.332935	80.088157
Var(3)	63.173473	17.453348	28.965539	97.381407

Fixed Effects Parameter Estimates

Term	Estimate	Std Error	DFDen	t Ratio	Prob>|t|	95% Lower	95% Upper
Intercept	21.593138	8.580838	30.9	2.52	0.0173	4.0890512	39.097225
repeat	-4.791689	0.8199182	27	-5.84	<.0001	-6.474023	-3.109356
clutchsize	0.185334	0.9196942	26	0.2	0.8419	-1.705124	2.0757924
total_mov	-0.003788	0.1556746	26.3	-0.02	0.9808	-0.323619	0.3160435
(total_mov-22.6552)*(repeat-2)	0.1590078	0.0793983	27	2	0.0553	-0.003904	0.3219196

Fixed Effects Tests

Source	Nparm	DFNum	DFDen	F Ratio	Prob > F
repeat	1	1	27	34.153583	<.0001
clutchsize	1	1	26	0.0406091	0.8419
total_mov	1	1	26.3	0.000592	0.9808
total_mov*repeat	1	1	27	4.0106489	0.0553

Fit Mixed

Actual by Predicted Plot

Fit Statistics

-2 Residual Log Likelihood	600.94243
-2 Log Likelihood	604.87321
AICc	630.39321
BIC	653.9982

Repeated Effects Covariance Parameter Estimates
Repeated Effect: repeat2

Covariance Parameter	Estimate	Std Error	95% Lower	95% Upper
Var(1)	125.77355	35.253287	56.678375	194.86872
Cov(2,1)	77.693768	27.768976	23.267576	132.11996
Var(2)	103.74262	28.927321	47.046111	160.43913
Cov(3,1)	53.271779	20.406358	13.276052	93.267506
Cov(3,2)	43.822062	18.134062	8.2799548	79.36417
Var(3)	63.597716	17.691543	28.92293	98.272503

Fixed Effects Parameter Estimates

Term	Estimate	Std Error	DFDen	t Ratio	Prob>|t|	95% Lower	95% Upper

Intercept	20.78792	8.9812777	27.8	2.31	0.0283	2.3833659	39.192473
repeat	-4.794811	0.8729323	27	-5.49	<.0001	-6.585911	-3.003712
clutchsize	-0.049555	0.9766676	25.3	-0.05	0.9599	-2.059638	1.9605273
total_mov	0.118298	0.1487883	26	0.8	0.4338	-0.187536	0.4241324
(clutchsize-8.31034)*(repeat-2)	0.3059819	0.5248966	27	0.58	0.5648	-0.771011	1.3829748

Fixed Effects Tests

Source	Nparm	DFNum	DFDen	F Ratio	Prob > F
repeat	1	1	27	30.170457	<.0001
clutchsize	1	1	25.3	0.0025745	0.9599
total_mov	1	1	26	0.6321457	0.4338
clutchsize*repeat	1	1	27	0.3398163	0.5648

Fit Mixed

Actual by Predicted Plot

Fit Statistics

-2 Residual Log Likelihood	599.43076
-2 Log Likelihood	605.45159
AICc	628.34633
BIC	650.11067

Repeated Effects Covariance Parameter Estimates
Repeated Effect: repeat2

Covariance Parameter	Estimate	Std Error	95% Lower	95% Upper
Var(1)	118.9128	32.300446	55.605088	182.22051
Cov(2,1)	70.968554	24.669357	22.617504	119.3196
Var(2)	97.150283	25.960455	46.268725	148.03184
Cov(3,1)	50.513494	19.429932	12.431527	88.59546
Cov(3,2)	41.198498	17.135933	7.6126868	74.784309
Var(3)	64.945437	17.587742	30.474096	99.416778

Fixed Effects Parameter Estimates

Term	Estimate	Std Error	DFDen	t Ratio	Prob>|t|	95% Lower	95% Upper
Intercept	22.938766	8.3288485	29	2.75	0.0101	5.9032115	39.97432
repeat	-4.794813	0.8729502	27	-5.49	<.0001	-6.585956	-3.00367
clutchsize	0.0138435	0.9459799	27	0.01	0.9884	-1.92714	1.9548269
(clutchsize-8.31034)*(repeat-2)	0.3059808	0.5249073	27	0.58	0.5648	-0.771038	1.3829998

Fixed Effects Tests

Source	Nparm	DFNum	DFDen	F Ratio	Prob > F
repeat	1	1	27	30.16924	<.0001
clutchsize	1	1	27	0.0002142	0.9884
clutchsize*repeat	1	1	27	0.3397998	0.5648

Fit Mixed

Actual by Predicted Plot

Fit Statistics

-2 Residual Log Likelihood	602.69183

-2 Log Likelihood	601.10894
AICc	624.00368
BIC	645.76803

Repeated Effects Covariance Parameter Estimates
Repeated Effect: repeat2

Covariance Parameter	Estimate	Std Error	95% Lower	95% Upper
Var(1)	117.73675	32.010016	54.998274	180.47523
Cov(2,1)	69.071785	24.44984	21.150979	116.99259
Var(2)	97.125137	26.200058	45.773967	148.47631
Cov(3,1)	53.027551	19.226936	15.343449	90.711652
Cov(3,2)	43.08182	16.950747	9.8589663	76.304674
Var(3)	61.147315	16.616945	28.578702	93.715929

Fixed Effects Parameter Estimates

Term	Estimate	Std Error	DFDen	t Ratio	Prob>|t|	95% Lower	95% Upper
Intercept	23.072299	4.3778893	31.7	5.27	<.0001	14.151205	31.993393
repeat	-4.791689	0.8199172	27	-5.84	<.0001	-6.47402	-3.109359
total_mov	-0.001093	0.152575	27	-0.01	0.9943	-0.314151	0.311965
(total_mov-22.6552)*(repeat-2)	0.1590078	0.0793982	27	2	0.0553	-0.003904	0.3219194

Fixed Effects Tests

Source	Nparm	DFNum	DFDen	F Ratio	Prob > F
repeat	1	1	27	34.153663	<.0001
total_mov	1	1	27	5.13E-05	0.9943
total_mov*repeat	1	1	27	4.0106595	0.0553

Fit Mixed

Actual by Predicted Plot

Fit Statistics

-2 Residual Log Likelihood	594.55127
-2 Log Likelihood	603.46583
AICc	628.98583
BIC	652.59082

Repeated Effects Covariance Parameter Estimates
Repeated Effect: repeat2

Covariance Parameter	Estimate	Std Error	95% Lower	95% Upper
Var(1)	112.30617	30.795873	51.947367	172.66497
Cov(2,1)	64.142572	23.295482	18.484267	109.80088
Var(2)	90.099228	24.521578	42.037818	138.16064
Cov(3,1)	47.529558	19.026892	10.237534	84.821582
Cov(3,2)	37.995752	16.706792	5.2510418	70.740462
Var(3)	65.584155	18.098906	30.110952	101.05736

Fixed Effects Parameter Estimates

Term	Estimate	Std Error	DFDen	t Ratio	Prob>|t|	95% Lower	95% Upper
Intercept	22.037517	8.1037815	28.2	2.72	0.0111	5.4420171	38.633016
repeat	-4.794813	0.8729471	27	-5.49	<.0001	-6.585949	-3.003676
age	2.5080402	1.8788754	26	1.33	0.1935	-1.353989	6.3700694
clutchsize	-0.387941	0.9645183	26.5	-0.4	0.6908	-2.368678	1.5927958
(clutchsize-8.31034)*(repeat-2)	0.3059809	0.5249054	27	0.58	0.5648	-0.771034	1.382996

Fixed Effects Tests

Source	Nparm	DFNum	DFDen	F Ratio	Prob > F
repeat	1	1	27	30.169455	<.0001
age	1	1	26	1.7818578	0.1935
clutchsize	1	1	26.5	0.1617747	0.6908
clutchsize*repeat	1	1	27	0.3398025	0.5648

Fit Mixed

Actual by Predicted Plot

Fit Statistics

-2 Residual Log Likelihood	598.31326
-2 Log Likelihood	599.56683
AICc	625.08683
BIC	648.69182

Repeated Effects Covariance Parameter Estimates
Repeated Effect: repeat2

Covariance Parameter	Estimate	Std Error	95% Lower	95% Upper
Var(1)	111.93586	30.772323	51.623218	172.24851
Cov(2,1)	62.767439	23.301924	17.096507	108.43837
Var(2)	90.307043	24.940706	41.424157	139.18993
Cov(3,1)	50.710788	19.006488	13.458756	87.96282
Cov(3,2)	40.269177	16.647976	7.6397432	72.898612
Var(3)	62.309601	17.293722	28.414528	96.204673

Fixed Effects Parameter Estimates

Term	Estimate	Std Error	DFDen	t Ratio	Prob>|t|	95% Lower	95% Upper
Intercept	20.203302	4.949186	33.3	4.08	0.0003	10.137359	30.269246
repeat	-4.791683	0.8198721	27	-5.84	<.0001	-6.473908	-3.109457
age	2.1499679	1.8029819	26	1.19	0.2438	-1.555936	5.855872
total_mov	-0.034925	0.150344	26.1	-0.23	0.8181	-0.343913	2.74E-01
(total_mov-22.6552)*(repeat-2)	0.1590226	0.0793938	27	2	0.0553	-0.003879	0.321924

Fixed Effects Tests

Source	Nparm	DFNum	DFDen	F Ratio	Prob > F
repeat	1	1	27	34.157325	<.0001
age	1	1	26	1.4219399	0.2438
total_mov	1	1	26.1	0.0539644	0.8181
total_mov*repeat	1	1	27	4.0118471	0.0553

Fit Mixed

Actual by Predicted Plot

Fit Statistics

-2 Residual Log Likelihood	594.55127
-2 Log Likelihood	603.46583
AICc	628.98583
BIC	652.59082

Repeated Effects Covariance Parameter Estimates
Repeated Effect: repeat2

Covariance Parameter	Estimate	Std Error	95% Lower	95% Upper
Var(1)	112.30617	30.795873	51.947367	172.66497
Cov(2,1)	64.142572	23.295482	18.484267	109.80088
Var(2)	90.099228	24.521578	42.037818	138.16064
Cov(3,1)	47.529558	19.026892	10.237534	84.821582
Cov(3,2)	37.995752	16.706792	5.2510418	70.740462
Var(3)	65.584155	18.098906	30.110952	101.05736

Fixed Effects Parameter Estimates

Term	Estimate	Std Error	DFDen	t Ratio	Prob>|t|	95% Lower	95% Upper
Intercept	22.037517	8.1037815	28.2	2.72	0.0111	5.4420171	38.633016
repeat	-4.794813	0.8729471	27	-5.49	<.0001	-6.585949	-3.003676
age	2.5080402	1.8788754	26	1.33	0.1935	-1.353989	6.3700694
clutchsize	-0.387941	0.9645183	26.5	-0.4	0.6908	-2.368678	1.5927958
(clutchsize-8.31034)*(repeat-2)	0.3059809	0.5249054	27	0.58	0.5648	-0.771034	1.382996

Fixed Effects Tests

Source	Nparm	DFNum	DFDen	F Ratio	Prob > F
repeat	1	1	27	30.169455	<.0001
age	1	1	26	1.7818578	0.1935
clutchsize	1	1	26.5	0.1617747	0.6908
clutchsize*repeat	1	1	27	0.3398025	0.5648

Fit Mixed

Actual by Predicted Plot

Fit Statistics

-2 Residual Log Likelihood	598.57428
-2 Log Likelihood	600.91841
AICc	626.43841
BIC	650.0434

Repeated Effects Covariance Parameter Estimates
Repeated Effect: repeat2

Covariance Parameter	Estimate	Std Error	95% Lower	95% Upper
Var(1)	118.16653	32.092251	55.266876	181.06619
Cov(2,1)	69.109503	24.44516	21.19787	117.02114
Var(2)	97.153922	25.962385	46.268582	148.03926
Cov(3,1)	52.579874	19.229531	14.890685	90.269062
Cov(3,2)	43.061623	16.976551	9.7881952	76.335051
Var(3)	61.570091	16.724661	28.790358	94.349825

Fixed Effects Parameter Estimates

Term	Estimate	Std Error	DFDen	t Ratio	Prob>|t|	95% Lower	95% Upper
Intercept	22.932586	8.3110669	28.7	2.76	0.01	5.9275893	39.937583
repeat	-4.791723	0.8295485	26.3	-5.78	<.0001	-6.496014	-3.087431
clutchsize	0.0138434	0.9460003	27	0.01	0.9884	-1.927189	1.9548754
(total_mov-22.6552)*(repeat-2)	0.1555037	0.0767461	26	2.03	0.0531	-0.00225	0.3132576
(clutchsize-8.31034)*(repeat-2)	0.2226409	0.5005917	26.3	0.44	0.6601	-0.805721	1.2510026

Fixed Effects Tests

Source	Nparm	DFNum	DFDen	F Ratio	Prob > F
repeat	1	1	26.3	33.365665	<.0001
clutchsize	1	1	27	0.0002141	0.9884
total_mov*repeat	1	1	26	4.1055268	0.0531
clutchsize*repeat	1	1	26.3	0.1978075	0.6601

Fit Mixed

Actual by Predicted Plot

Fit Statistics

-2 Residual Log Likelihood	599.15902
-2 Log Likelihood	601.06405
AICc	623.95879
BIC	645.72313

Repeated Effects Covariance Parameter Estimates
Repeated Effect: repeat2

Covariance Parameter	Estimate	Std Error	95% Lower	95% Upper
Var(1)	116.56176	31.315881	55.18376	177.93976
Cov(2,1)	68.432467	24.072609	21.251021	115.61391
Var(2)	96.897808	25.846733	46.239141	147.55647
Cov(3,1)	52.816806	19.079475	15.421722	90.21189
Cov(3,2)	43.182342	16.955744	9.9496935	76.41499
Var(3)	61.500172	16.694882	28.778805	94.221539

Fixed Effects Parameter Estimates

Term	Estimate	Std Error	DFDen	t Ratio	Prob>|t|	95% Lower	95% Upper
Intercept	21.564729	7.9421666	31.7	2.72	0.0106	5.3815696	37.747888
repeat	-4.791704	0.8174865	27.3	-5.86	<.0001	-6.468276	-3.115133
clutchsize	0.1784391	0.9001276	27	0.2	0.8443	-1.66837	2.0252482
(total_mov-22.6552)*(repeat-2)	0.1582656	0.0754707	27	2.1	0.0455	0.0034062	0.3131249

Fixed Effects Tests

Source	Nparm	DFNum	DFDen	F Ratio	Prob > F
repeat	1	1	27.3	34.357285	<.0001
clutchsize	1	1	27	0.0392981	0.8443
total_mov*repeat	1	1	27	4.3976071	0.0455

Fit Mixed

Actual by Predicted Plot

Fit Statistics

-2 Residual Log Likelihood	602.2266
-2 Log Likelihood	600.9186
AICc	626.4386
BIC	650.04359

Repeated Effects Covariance Parameter Estimates
Repeated Effect: repeat2

Covariance Parameter	Estimate	Std Error	95% Lower	95% Upper
Var(1)	118.18089	32.142065	55.183603	181.17818
Cov(2,1)	69.098424	24.485187	21.10834	117.08851
Var(2)	97.125681	26.200349	45.773941	148.47742
Cov(3,1)	52.590727	19.27614	14.810187	90.371268
Cov(3,2)	43.055491	16.993783	9.7482888	76.362693
Var(3)	61.578168	16.755425	28.738139	94.418198

Fixed Effects Parameter Estimates

Term	Estimate	Std Error	DFDen	t Ratio	Prob>|t|	95% Lower	95% Upper
Intercept	23.072358	4.3854537	31.8	5.26	<.0001	14.136915	32.0078
repeat	-4.791718	0.8297375	26.3	-5.77	<.0001	-6.496398	-3.087038
total_mov	-0.001093	0.1525754	27	-0.01	0.9943	-0.314152	0.3119658
(total_mov-22.6552)*(repeat-2)	0.1557291	0.080636	26.3	1.93	0.0643	-0.009921	0.3213791
(clutchsize-8.31034)*(repeat-2)	0.2254955	0.4765507	26	0.47	0.64	-0.754068	1.2050594

Fixed Effects Tests

Source	Nparm	DFNum	DFDen	F Ratio	Prob > F
repeat	1	1	26.3	33.3504	<.0001
total_mov	1	1	27	5.13E-05	0.9943
total_mov*repeat	1	1	26.3	3.7297606	0.0643
clutchsize*repeat	1	1	26	0.2239019	0.64

Fit Mixed

Actual by Predicted Plot

Fit Statistics

-2 Residual Log Likelihood	602.64627
-2 Log Likelihood	604.7895
AICc	627.68423
BIC	649.44858

Repeated Effects Covariance Parameter Estimates
Repeated Effect: repeat2

Covariance Parameter	Estimate	Std Error	95% Lower	95% Upper
Var(1)	121.70033	33.474493	56.091526	187.30913
Cov(2,1)	74.317418	26.368002	22.637083	125.99775
Var(2)	100.93544	27.849679	46.351076	155.51981
Cov(3,1)	50.730427	19.394049	12.71879	88.742065
Cov(3,2)	41.725451	17.282801	7.8517829	75.599118
Var(3)	62.092491	17.032321	28.709755	95.475227

Fixed Effects Parameter Estimates

Term	Estimate	Std Error	DFDen	t Ratio	Prob>|t|	95% Lower	95% Upper
Intercept	20.482668	4.3256792	41.7	4.74	<.0001	11.751446	29.21389
repeat	-4.794814	0.8703103	27.3	-5.51	<.0001	-6.579644	-3.009984
total_mov	0.1135903	0.1458354	27	0.78	0.4428	-0.185623	0.4128033
(clutchsize-8.31034)*(repeat-2)	0.2954724	0.4952865	26	0.6	0.556	-0.72261	1.3135546

Fixed Effects Tests

Source	Nparm	DFNum	DFDen	F Ratio	Prob > F
repeat	1	1	27.3	30.352559	<.0001
total_mov	1	1	27	0.6066759	0.4428
clutchsize*repeat	1	1	26	0.3558942	0.556

Fit Mixed

Actual by Predicted Plot

Fit Statistics

-2 Residual Log Likelihood	600.21637
-2 Log Likelihood	600.86395
AICc	623.75868
BIC	645.52303

Repeated Effects Covariance Parameter Estimates
Repeated Effect: repeat2

Covariance Parameter	Estimate	Std Error	95% Lower	95% Upper
Var(1)	114.67898	30.636238	54.633055	174.7249
Cov(2,1)	66.215981	23.301033	20.546796	111.88517
Var(2)	94.753164	25.090341	45.576999	143.92933
Cov(3,1)	50.28478	18.339808	14.339417	86.230142
Cov(3,2)	41.157226	16.222692	9.3613334	72.953118
Var(3)	60.059179	16.072995	28.556688	91.56167

Fixed Effects Parameter Estimates

Term	Estimate	Std Error	DFDen	t Ratio	Prob>|t|	95% Lower	95% Upper
Intercept	23.047626	2.6632482	27.8	8.65	<.0001	17.590175	28.505077
repeat	-4.791722	0.8272766	26.5	-5.79	<.0001	-6.490587	-3.092858
(total_mov-22.6552)*(repeat-2)	0.1555038	0.0766569	26	2.03	0.0529	-0.002067	0.3130742
(clutchsize-8.31034)*(repeat-2)	0.2254956	0.4759969	26	0.47	0.6396	-0.752929	1.2039199

Fixed Effects Tests

Source	Nparm	DFNum	DFDen	F Ratio	Prob > F
repeat	1	1	26.5	33.549169	<.0001
total_mov*repeat	1	1	26	4.1150868	0.0529
clutchsize*repeat	1	1	26	0.2244232	0.6396

Fit Mixed

Actual by Predicted Plot

Fit Statistics

-2 Residual Log Likelihood	601.07285
-2 Log Likelihood	605.39726
AICc	625.73492
BIC	645.59043

Repeated Effects Covariance Parameter Estimates
Repeated Effect: repeat2

Covariance Parameter	Estimate	Std Error	95% Lower	95% Upper
Var(1)	115.42895	30.832902	54.997578	175.86033
Cov(2,1)	68.077722	23.52489	21.969785	114.18566
Var(2)	94.75367	25.09061	45.576978	143.93036
Cov(3,1)	48.221676	18.547678	11.868895	84.574457
Cov(3,2)	39.296635	16.384408	7.1837855	71.409484
Var(3)	63.438023	16.936378	30.243332	96.632713

Fixed Effects Parameter Estimates

Term	Estimate	Std Error	DFDen	t Ratio	Prob>|t|	95% Lower	95% Upper
Intercept	23.053806	2.7187573	27.9	8.48	<.0001	17.483511	28.624102
repeat	-4.794812	0.8707977	27.2	-5.51	<.0001	-6.580794	-3.008831
(clutchsize-8.31034)*(repeat-2)	0.30884	0.5032101	27	0.61	0.5445	-0.723662	1.3413418

Fixed Effects Tests

Source	Nparm	DFNum	DFDen	F Ratio	Prob > F
repeat	1	1	27.2	30.318569	<.0001
clutchsize*repeat	1	1	27	0.3766763	0.5445

Fit Mixed

Actual by Predicted Plot

Fit Statistics

-2 Residual Log Likelihood	600.6816
-2 Log Likelihood	601.05436
AICc	621.39202
BIC	641.24753

Repeated Effects Covariance Parameter Estimates
Repeated Effect: repeat2

Covariance Parameter	Estimate	Std Error	95% Lower	95% Upper
Var(1)	114.23641	30.505735	54.446272	174.02656
Cov(2,1)	66.190231	23.266307	20.589107	111.79136
Var(2)	94.753671	25.090611	45.576978	143.93036
Cov(3,1)	50.72227	18.288999	14.876491	86.568049
Cov(3,2)	41.18411	16.180199	9.4715029	72.896717
Var(3)	59.629307	15.935203	28.396883	90.86173

Fixed Effects Parameter Estimates

Term	Estimate	Std Error	DFDen	t Ratio	Prob>|t|	95% Lower	95% Upper
Intercept	23.047569	2.6508068	27.9	8.69	<.0001	17.616698	28.478441
repeat	-4.791694	0.8174346	27.3	-5.86	<.0001	-6.468144	-3.115244
(total_mov-22.6552)*(repeat-2)	0.1587825	0.0752977	27	2.11	0.0444	0.0042844	0.3132807

Fixed Effects Tests

Source	Nparm	DFNum	DFDen	F Ratio	Prob > F
repeat	1	1	27.3	34.361499	<.0001
total_mov*repeat	1	1	27	4.4467424	0.0444

Min	621.39202
Min Bic	641.24753
